# Zwitterionic poly-carboxybetaine-dexamethasone conjugates do not alleviate cartilage degeneration and synovitis in the collagenase-induced osteoarthritis model in rats

**DOI:** 10.1038/s41598-025-93247-3

**Published:** 2025-07-01

**Authors:** Patrick Weber, Maryam Asadikorayem, Shipin Zhang, David Fercher, Kajetana Bevc, Sami Kauppinen, Tuomas Frondelius, Tianqi Zhang, Marina Fonti, Gonçalo Barreto, Mikko A.J. Finnilä, Marcy Zenobi-Wong

**Affiliations:** 1 Tissue Engineering + Biofabrication Laboratory, Department of Health Sciences and Technology, ETH Zürich, Otto-Stern-Weg 7, Zürich, 8093 Switzerland; 2 Research Unit of Health Sciences and Technology, University of Oulu, Aapistie 5A, Oulu, 90220 Finland; 3 Clinicum, Faculty of Medicine, University of Helsinki and Helsinki University Hospital, Haartmaninkatu 8, Helsinki, 00290 Finland; 4 Biocenter Oulu, University of Oulu, Aapistie 5A, Oulu, 90220 Finland

**Keywords:** Zwitterionic polymers, Dexamethasone, CIOA, Computed tomography, Synovitis, Tissues, Bioinspired materials, Tissue engineering

## Abstract

Osteoarthritis is a degenerative joint disease for which there is yet to be a disease-modifying drug available in clinics. New drug candidates often fail due to a combination of poor pharmacokinetics as well as an inability to address the complex, multifactorial nature of osteoarthritis. To address these issues, we developed a zwitterionic poly-carboxybetaine acrylamide-dexamethasone (pCBAA-DEX) conjugate showing good cartilage penetration as well as anti-inflammatory and lubricating properties in previous in vitro studies. Here, we investigate the therapeutic potential of pCBAA-DEX in the collagenase-induced osteoarthritis (CIOA) model in rats. Upon induction of the model, animals received one-time, unilateral injections of either saline, DEX or pCBAA-DEX on day 4 (*N* = 8). On day 70, joint tissues were harvested and analyzed. While pCBAA-DEX achieved ~ 50% cartilage retention at the terminal timepoint, it did not prevent cartilage degeneration, synovial inflammation and synovial fibrosis, nor did DEX alone. Nevertheless, DEX and pCBAA-DEX slightly decreased the fibrosis levels in the synovium with DEX also decreasing the number of synovial lining layers. For the cartilage, DEX did not cause any notable differences, instead we observed an increase in cartilage degeneration in the pCBAA-DEX group. These findings challenge the previous in vitro results and motivate a substantial redesign of these conjugates and associated in vitro methods to reconsider them for the treatment of osteoarthritis.

**Supplementary Information:**

The online version contains supplementary material available at 10.1038/s41598-025-93247-3.

## Introduction

Osteoarthritis (OA) is one of the main causes of pain and disability worldwide^1^ and no disease-modifying drug is yet available in clinical practice^2^. Currently, the pharmacological treatment of OA mainly involves the use of intra-articularly injected, short-acting anti-inflammatory drugs such as glucocorticoids (GCs) or non-steroidal anti-inflammatory drugs as well as hyaluronic acid(HA)-based viscosupplements^3,4^ all of which fail to prevent progression of the disease in the long run^5–8^. For GCs specifically, several preclinical studies in vitro and in vivo have suggested a potential disease-modifying effect, however, these studies all rely on sustained-release platforms to ensure a continuous release of GCs at a low dose into the joint cavity over several weeks^9–11^. In a clinical setting, GCs are injected in an untargeted way that results in an extremely poor pharmacokinetic profile with a joint half-life of only a few hours at most^12^. Moreover, GCs are typically injected at concentrations that are up to five orders of magnitude above the therapeutic concentration that has been linked to detrimental effects on cartilage health in several clinical studies^6,13–15^.

**Fig. 1:**
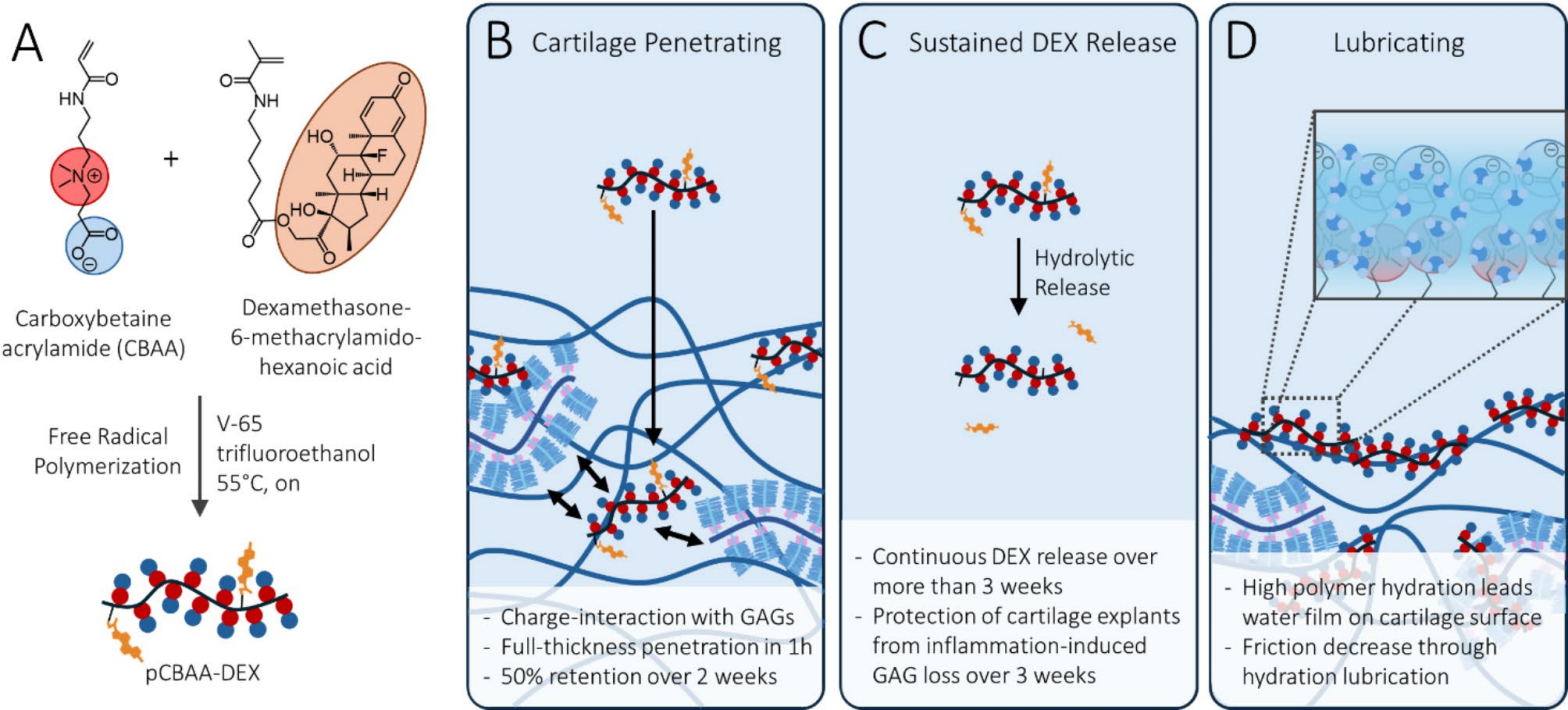
In vitro properties of the pCBAA-DEX polymer-drug conjugate: (**A**) pCBAA-DEX was synthesized via co-polymerization of CBAA and a DEX comonomer via free radical polymerization. (**B**) Previous studies have found good cartilage penetration and retention properties for pCBAA polymers, which was attributed to a small net positive polymer charge that enabled charge-interactions with the anionic GAGs. (**C**) pCBAA-DEX was found to continuously release DEX via hydrolytic cleavage of the linking ester over 3 weeks. In an inflammatory OA model, cartilage explants treated with pCBAA-DEX were protected from GAG loss over three weeks. (**D**) At the cartilage surface, pCBAA polymers increased the hydrophilicity and decreased friction through the hydration lubrication mechanism.

To address these issues, we have recently developed a conjugate of zwitterionic poly-carboxybetaine acrylamide (pCBAA) and the GC dexamethasone (DEX, Fig. 1)^16^. Zwitterionic polymers are highly hydrophilic materials that have recently gained popularity in biomedical engineering due to their reported non-fouling^17,18^ and lubricating properties^19,20^. This has resulted in a variety of applications including the development of non-fouling hydrogels to prevent foreign body reactions to implants^18^, polymer-protein conjugates to increase in vivo stability^21^ and surface modifications to reduce friction^20^. Of all zwitterionic polymer types, pCBAA polymers are known to have particularly high hydration levels, which motivated our choice of this particular chemistry^22^. Due to the high intramolecular density and proximity of the pCBAA side chains, we found that the protonated state of the carboxylic acids is stabilized through hydrogen bonds, thereby resulting in a small net positive charge of the polymer at physiological pH. The anionic charge of the cartilage extracellular matrix (ECM) thus allows for excellent penetration and retention kinetics and further motivated its use as a drug carrier. The resulting polymer-drug conjugate (Fig. 1) showed sustained release of DEX over up to three weeks and protected cartilage explants from the loss of glycosaminoglycans (GAGs) when treated with the inflammatory cytokine interleukin-1β (IL-1β). In another study^23^, we also investigated the lubrication properties of pCBAA as their chemistry mimics that of surface- active phospholipids which are one of the main boundary lubricants in synovial joints^24–26^. As these phospholipids are known to be depleted in OA patients^27^, we hypothesized that we could restore boundary lubrication by application of pCBAA. We found that the treatment with pCBAA not only restored the boundary lubrication of IL-1β-treated cartilage explants at the surface, but also, through its full-thickness penetration, decreased tissue permeability to potentially enable a more sustained weeping lubrication mechanism.

The goal of this study was to investigate the therapeutic potential of our pCBAA-DEX conjugate in the collagenase-induced OA (CIOA) model which triggers a relatively mild OA-like phenotype that mimics human post-traumatic OA^28,29^. In this model, initially described by *van der Kraan et al.* in 1990, collagenase is injected into the knee joint which amongst others weakens the ligaments, thereby causing persistent joint destabilization similar to that in surgically-induced animal models of OA^30^. Over time, this destabilization leads to continuous cartilage degeneration and a decrease in subchondral bone thickness and the development of osteophytes^30,31^. In comparison to surgically-induced OA models, the injected collagenase in the CIOA model also triggers a strong inflammatory response in the synovium that subsequently undergoes fibrotic remodeling and retains signs of low-grade inflammation in the long term^28,32,33^. Similar to human OA, the mechanical and inflammatory factors are closely intertwined which makes the CIOA model particularly attractive for OA research^34,35^.

Upon unilateral, intraarticular injection of collagenase on days 0 and 2 of the study, the animals received a single injection of either saline, DEX or pCBAA-DEX on day 4. This treatment scheme was chosen based on previous experience with this model to best mimic the case of post-traumatic OA which is typically initiated by an acute phase of local, trauma-induced inflammation and requires rapid treatment^28^. Similarly, a scheme with repeated drug administration was avoided to provide a more challenging and clinically relevant setting. Considering the dose-dependent lubrication properties, the pCBAA-DEX conjugate was injected at a concentration of 10 mg/mL and the degree of DEX loading was controlled such that both DEX-containing treatments contained 20 µM of the drug. This DEX concentration is around 2 orders of magnitude below the clinically used concentrations^13^ and should allow for the continuous release of DEX in the mid-to-high nanomolar range for around three weeks^16^. Upon euthanasia on day 70, analysis was performed on all synovium, cartilage and bone tissues in accordance with the multi-tissue nature of OA. By performing multiple, complementary analysis techniques on the collected tissues, we aimed at decoupling the different mechanisms driving the CIOA model to ultimately get more concrete insights on how the cartilage homing, anti-inflammatory and lubricating properties of pCBAA-DEX were effective in vivo.

## Materials and methods

### Chemicals

Unless otherwise stated, all chemicals were purchased from Sigma-Aldrich (CH).

### CBAA monomer synthesis

CBAA was synthesized as previously reported^16^: 14.9 g freshly distilled N-[3-(Dimethylamino)propyl] acrylamide (95 mmol, TCI Chemicals, JPN) was dissolved in 100 mL of dry THF. After cooling to -10 °C, 9.6 g of β-propiolactone (8.4 mL, 134 mmol, 1.4 eq., Acros Organics, USA) in 25 mL of dry THF was added dropwise over 40 min. After 4 h of continuous stirring, the reaction mixture was kept at -20 °C overnight. The white precipitate was separated from the reaction mixture via filtration with a fritted-glass filter (S4 porosity) and washed with three volumes of cold diethylether. After drying under vacuum, CBAA was isolated as a white solid (13.0 g, 57 mmol, 60% yield)^1^. H-NMR (Bruker 400 MHz in D_2_O): δ (ppm) 6.32 (dd, 1 H, CHH = C*H*), 6.2 (dd, 1 H, CH*H* = CH), 5.87 (dd, 1 H, C*H*H = CH), 3.66 (t, 2 H, N-C*H*_2_-CH_2_-COO), 3.48 (m, 4 H, NH-C*H*_2_-CH_2_-C*H*_2_), 3.17 (s, 6 H, N-(C*H*_3_)_2_), 2.75 (t, 2 H, C*H*_2_-COO), 1.98 (dt, 2 H, NH-CH_2_-C*H*_2_-CH_2_).

### DEX-MAHAc synthesis

DEX-MAHAc was synthesized in two steps as previously reported^16^.

#### 6-methacrylamidohexanoic acid

3.9 g of 6-aminocaproic acid (30 mmol) was dissolved in 3 mL of dH_2_O and mixed with 1.2 g of NaOH dissolved in 3 mL of dH_2_O (30 mmol, 1 eq.). The mixture was cooled to 0 °C, after which 2.9 mL of neat methacryloyl chloride (3.1 g, 30 mmol, 1 eq.) and a solution of 1.2 g of NaOH in 6 mL of dH_2_O (30 mmol, 1 eq.) were added dropwise simultaneously over 30 min. The reaction mixture was stirred at room temperature for 1 h and acidified with concentrated HCl to pH 2. The crude was extracted with dichloromethane, dried over MgSO_4_, and recrystallized from ethyl acetate, to isolate the product as a white crystalline solid (3.5 g, 18 mmol, 60% yield)^1^. H-NMR (Bruker 400 MHz in DMSO-d6): δ (ppm) 12.00 (s, 1 H, (CH_2_)_5_-COO*H*), 7.88 (s, 1 H, *H*N-C(= O)-C(-CH_3_) = CH_2_), 5.62 (s, 1 H, C(= O)-C(-CH_3_) = C*H*H), 5.29 (s, 1 H, C(= O)-C(-CH_3_) = CH*H*), 3.09 (q, 2 H, NH-C*H*_2_-(CH_2_)_4_-COOH), 2.20 (t, 2 H, NH-(CH_2_)_4_-C*H*_2_-COOH), 1.85 (s, 3 H, C(= O)-C(-C*H*_3_) = CH_2_), 1.21–1.56 (m, 6 H, NH-CH_2_-(C*H*_2_)_3_-CH_2_-COOH.

#### Dexamethasone-6-methacrylamidohexanoic acid (DEX-MAHAc)

160 mg of Dexamethasone (0.41 mmol, ABCR, GER), 100 mg of 6-methacrylamidohexanoic acid (0.5 mmol, 1.2 eq.), 12 mg of 4-dimethylaminopyridine (0.10 mmol, 0.25 eq.) and 160 mg of N, N’-dicyclohexylcarbodiimide (0.78 mmol, 1.9 eq.) were dissolved in 2 mL of DMF and stirred at room temperature overnight. The white suspension was concentrated under reduced pressure and purified with flash column chromatography (SiO_2_, ethyl acetate/hexane 2:1 to pure ethyl acetate) to isolate the product as a colorless oil (178 mg, 0.31 mmol, 76% yield)^1^. H-NMR (Bruker 400 MHz in CDCl_3_): δ (ppm) Methacrylic protons: 5.67 (s, 1 H, C(= O)-C(-CH_3_) = C*H*H), 5.31 (s, 1 H, C(= O)-C(-CH_3_) = CH*H*), 1.93 (s, 3 H, C(= O)-C(-C*H*_3_) = CH_2_); DEX A-ring protons: 7.23 (d, 1 H), 6.33 (dd, 1 H), 6.11 (d, 1 H); Linking protons: 4.82–4.95 (dd, 2 H, DEX-C*H*_2_-O-C(= O)-CH_2_-MAHAc), 2.31–2.50 (m, 2 H, DEX-CH_2_-O-C(= O)-C*H*_2_-MAHAc). (LC-MS (ESI, +, m/z): found: 575 theoretical: 574.31.

### pCBAA-DEX synthesis

410 mg of CBAA (1.80 mmol), 0.52 mg of DEX-MAHAc (0.90 µmol, 0.05 mol%), 0.59 mg of acryloxyethyl thiocarbamoyl rhodamine B (0.90 µmol, 0.05 mol%) and 2.24 mg of V-65 (9.0 µmol, 0.5 mol%, WAKO Fujifilm, GER) were dissolved in 2 mL of trifluoroethanol. After purging with N_2_ for 10 min, the solution was stirred under protection from light at 55 °C overnight. The reaction mixture was diluted with dH_2_O, transferred to seamless cellulose dialysis membranes (12.4 kDa MWCO), dialyzed against 0.1 M NaCl (3 × 12 h) and dH_2_O (3 × 12 h) and lyophilized to yield the purified polymer as a pink, fluffy solid (350 mg, 85% yield). With 1 H-NMR, using the DEX A-ring protons, the degree of substitution was determined at 0.045 mol%.

### Animal experimentation

#### Animals

The animal study was approved by the Veterinary Office of the Canton Zürich (License No. ZH088/2023) and was conducted in accordance with the Swiss Animal Welfare Act. Moreover, the study was in compliance with the ARRIVE guidelines. 28 outbred, skeletally-mature Wistar rats (14 males (534 g ± 45 g), 14 females (268 g ± 19 g), 6 months old from Janvier Labs, FRA) were acclimatized to the new housing environment for 4 weeks after arrival. Animals were housed in groups (females: 3 animals/cage, males: 2 animals/cage) in individually ventilated cages (1500U SEAL SAFE, Tecniplast, AUS, 1500 cm^2^). Animals were held in a standard laboratory animal environment (21 ± 3 °C and 12/12 h light–dark cycle), received acidified tap water (pH 2.5-3.0) and standard laboratory rodent diet (3437, KLIBA NAFAG, CH) with specific opportunistic pathogen-free hygiene. Paper tissues and gnawing wood were provided as enrichment to promote animal wellbeing and movement in the cage. Each animal represents an experimental unit.

#### Intra-articular injections

Animals were randomly allocated to the study groups ensuring equal distribution amongst the two sexes and that all animals in one cage were in a different group. The only exceptions were the 4 naïve animals (2 males/2 females) where both animals per sex were held in the same cage. For the three other groups (saline, DEX, pCBAA-DEX), 8 animals (4 males/4 females) were allocated per group. Starting from day − 2 till day 5 of the study, all animals except the naïve received 25 mg/L tramadol in the drinking water as an analgesic. On day 0, the rats were anesthetized with isoflurane (5%, then 1.5% to maintain anesthesia) and their hindlimbs were shaved and disinfected (Kodan Tincture forte, Schülke, CH). The animals then received an intra-articular injection of 25 µL of 500 U collagenase into a randomized knee joint at 90° flexion through the patellar tendon using a 30 G x 8 mm insulin syringes (BD, USA). After the injection, the knees were gently articulated for 60 s to ensure an even distribution of the collagenase within the joint. On day 2, the procedure was repeated and on day 4, the animals received 25 µL of either saline, 20 µM DEX or 10 mg/mL pCBAA-DEX (both in saline) following the same procedure as for the collagenase. Note that the collagenase used in this study was from a low-activity batch (Stemcell Technologies, GER) and its enzymatic activity verified with a colorimetric assay (MAK293, Sigma-Aldrich, Table S1). On day 70, the rats were euthanized with CO_2_ and various tissues were harvested for analysis.

### Tissue harvest

The explanted joints were dissected to separate femur and tibia with the peripatellar joint capsule containing the patella, parts of the synovium and the patellar tendon still connected to the tibial tuberosity (Fig. S1). Ligaments and menisci were cut away to expose the cartilage. Together with some of the internal organs (heart, kidney, liver, lung and spleen), femur and tibia were taken for fluorescence stereomicroscopy immediately.

### Fluorescence stereomicroscopy

To assess the polymer distribution, internal organs, tibia and femur were imaged on a Leica M205 FA fluorescence stereomicroscope (Leica Microsystems, GER) using the rhodamine B channel. After imaging, the peripatellar joint capsule was removed from the tibia and all tissues were fixed in 4% paraformaldehyde. To estimate the fluorescence retention compared to day 4, we used fresh rat cadavers from a different study and injected them with 25 µL of 10 mg/mL pCBAA-DEX analogously as above. After 1.5 h of incubation at room temperature, the joints (*N* = 6) were explanted, dissected and imaged with the same settings on the fluorescence microscope. Fluorescence intensities were quantified in 4 separate regions (2x femoral condyle, femoral ligaments and fat pad) per joint with QuPath v0.4.2.

### X-ray Micro-Computed tomography (µCT)

The fixed femur and tibia were dehydrated to 70% ethanol, stained with 1% phosphotungstic acid (in 70% ethanol) for 24 h and imaged with a µCT 45 device (Scanco Medical, CH). The scans were acquired at 70 kV, 57 µA and 4 W using an 0.5 mm aluminum filter (1750 projections/180°, 2.5 s integration time per projection, 4.5 μm voxel size). The cartilage roughness score (CRS) was calculated as described elsewhere^36^. Briefly, a custom MatLab script^37^ was used to calculate the local surface orientations in a 28 μm^2^ neighborhood across the entire cartilage surface. Additionally, a smooth reference surface was constructed for each condyle using an iterative 5th -degree polynomial fit. The CRS then represents the angular difference between the local surface orientation and the reference surface averaged across the whole condyle. An increase in CRS therefore suggests a rougher cartilage surface, indicative of cartilage damage. See Figs. S2 and S3 for the full µCT dataset.

Using the same CT datasets, binary bone masks were extracted for femur and tibia and the local subchondral bone plate thickness measured with BoneJ in Fiji ImageJ v1.51n. The trabecular bone analysis was performed on the medullary volume that was isolated by identification of the subchondral bone and growth plate to create an ROI of the entire tissue volume and eroding it with a 315 μm circular kernel. Trabecular bone parameters were calculated with CTAn 1.20.3.0 (Bruker, USA). Both trabecular and subchondral bone parameters were averaged per condyle.

### Histology

The fixed peripatellar joint capsules, femurs and tibias were decalcified in aqueous 10% NH_4_-DETA solution (joint capsules: 7 days, femur/tibia: 10 days), dehydrated to 70% ethanol, paraffinized on a Milestone Logos J device (Milestone, ITA) and embedded in paraffin blocks (Fig. S1). 5 μm serial sections were prepared with a microtome (HM 325, Microm, GER) in transverse (joint capsule) and coronal (femur/tibia) orientation, respectively. For each tissue, two sections from the center of the tissue with > 200 μm spacing were selected and rehydrated for histological staining.

#### Hematoxylin and Eosin (H&E)

Rehydrated joint capsule sections were incubated with Gill No. 3 hematoxylin (Merck, GER) for 5 min, washed with water, incubated with 0.1% Na_2_CO_3_ for 40 s, and counterstained in 0.25% Eosin Y (in 80% ethanol) for 1 min. Finally, the sections were washed in 100% ethanol, cleared in xylene and coverslipped with Eukitt mounting medium. See Figs. S4–S7 for the full dataset.

#### Masson’s trichrome

Rehydrated joint capsule sections were stained with the trichrome stain kit (Abcam, UK) following manufacturer instructions and coverslipped with Eukitt mounting medium. See Figs. S8-S11 for the full dataset.

#### Safranin O

Rehydrated femur/tibia sections were stained with aqueous 0.5% safranin O for 16 min, washed with water, dehydrated to xylene and coverslipped with Eukitt mounting medium. See Fig. S12-S19 for full dataset.

### Quantitative histology analysis

#### Cell density

The cell density in synovia was quantified with QuPath v0.4.2 using the H&E sections. The regions of interest (ROIs) were drawn manually, with the borders being represented by the synovial lining, patella, fibrous membrane and the width of intact full-thickness tissue (Fig. S20). The cell number within the ROIs was calculated running QuPath’s built-in cell detection tool on the hematoxylin channel (background radius: 8.0 μm, sigma: 1.5 μm, min area: 12.0 μm, max area: 400 μm, threshold: 0.2, max background 2.0). For the detection of adipocytes, a pixel classifier was trained to extract the adipocyte areas as a binary mask on which particle analysis was run with the maximum area set to 800 μm^2^ and a minimum solidity of 0.75.

#### Masson’s trichrome color Deconvolution

To assess the density of collagens and other ECM components, the blue and red staining intensities were quantified in sections stained with Masson’s trichrome. Analogous to 2.9.1, ROIs were drawn manually in QuPath v0.4.2. The images were color deconvoluted (channel 1: 0.74977, 0.61536, 0.24328, channel 2: 0.09684, 0.74866, 0.65584, background: 249, 247, 247) and the mean intensity of channels 1 and 2 extracted for each ROI.

### Histology grading

Histopathological gradings of synovia and cartilage were performed as recommended by the Osteoarthritis Research Society International (OARSI)^38^: For the synovium, the “synovitis score” was applied and for the cartilage, the “cartilage degeneration score”(CDS), “total cartilage degeneration width” (TCDW) and “significant cartilage degeneration width” (SCDW). Note that the TCDW and SCDW describe how much of the cartilage surface (TCDW) and middle zone (SCDW) show any sign of cartilage degeneration (Fig. S21). All assessments were performed by three blinded graders and the results reported as the average of all graders. The agreement between the graders was assessed by calculation of the intra-class correlation (ICC (IBM SPSS Statistics v28)), which was above 0.85 for all analyses (synovitis: 0.918, CDS: 0.927, TCDW: 0.863, SCDW: 0.867).

### Statistical analysis

**Fig. 2:**
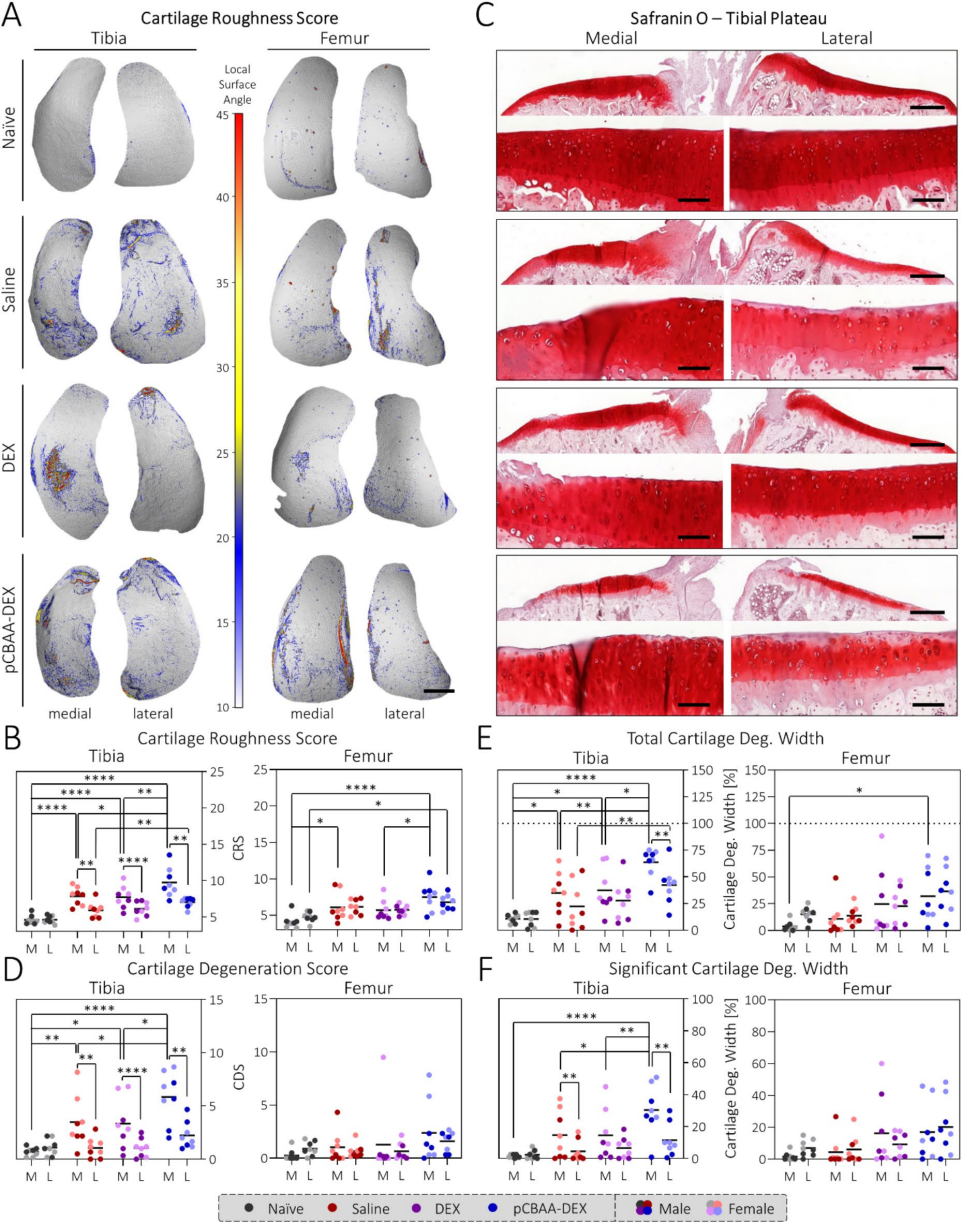
DEX and pCBAA-DEX do not prevent cartilage degeneration: (**A**) Representative computed tomography surface roughness maps for the four study groups. Scale bar: 1 mm. (**B**) Calculation of the CRS reveals no reduction in surface roughness upon treatment with either DEX or pCBAA-DEX. The latter even causes an increase in CRS in the tibia. (**C**) Representative Safranin O staining of the tibial plateaus of our four study groups. Scale bars: 500 μm (top), 100 μm (bottom). (**D**) Grading of the cartilage with the OARSI cartilage degeneration score^38^ matched the CRS results, as did the assessment of the total cartilage degeneration width (**E**) and the significant cartilage degeneration width (**F**). M: medial, L: lateral, *N* = 8. See Figures S2/3 and S12-19 for the full CRS and histology dataset. Note that all data comparisons without asterisks were statistically non-significant.

All statistical analysis was performed with GraphPad Prism v. 10.1.2 (GraphPad, USA). Normality was assessed with a Shapiro-Wilk test and confirmed for the majority (> 75%) of the collected data. Two-way analysis of variance (ANOVA) was performed with a Tukey’s multiple comparisons test for the data shown in Fig. 2 (factors: treatment, medial/lateral). For the data in all other figures, a one-way ANOVA was performed. Statistical significance is indicated with asterisks: * *p* < 0.05, ** *p* < 0.01, *** *p* < 0.001, **** *p* < 0.0001.

## Results

### No prevention of cartilage degeneration upon pCBAA-DEX treatment

To assess cartilage degeneration, we employed both the cartilage roughness score (CRS) as described by *Kauppinen et al.*^36^, and histopathological grading^38^. The CRS is based on a method in which the cartilage surface is first visualized with micro-computed tomography using a contrast agent and the degree of surface degeneration or roughness is then assessed by calculating the local surface angles on the cartilage tissue with respect to a smooth reference surface. The average value across the entire cartilage surface then represents the CRS. In comparison to conventional histopathological gradings, the CRS method has several advantages such as its objectivity and its ability to evaluate the entire cartilage surface and not just single sections. *Kauppinen et al.* estimate that up to 16 histological sections per joint would be necessary to reach a similar accuracy with histology-based assessments. On the other hand, histopathological grading goes beyond mere surface fibrillation and also includes additional features of cartilage degeneration such as cell death and ECM density, thereby complementing the CRS method.

Using CRS analysis, the effect of the collagenase injection was clearly visible as the saline group showed systematically higher roughness scores compared to the naïve animals (Fig. 2A, B). As described previously^28^, this was particularly pronounced for the medial tibia, where the saline group reached a CRS of 7.83 ± 1.24 compared to 4.60 ± 0.62 for the naïve animals (Fig. 2B). Treatments with DEX (7.72 ± 1.52) or pCBAA-DEX (9.73 ± 2.03) were both not able to prevent the increase in surface roughness; in fact, the value for pCBAA-DEX is even increased compared to the saline group. These findings were furthermore confirmed by the histopathological scoring where all three performed gradings (cartilage degeneration score (CDS), total cartilage degeneration width (TCDW) and significant cartilage degeneration width (SCDW)) mirrored the general trends found by the CRS (Fig. 2C-F). As expected, the average standard error within the groups was decreased for the volumetric CRS (18 ± 6%) compared to the section-based CDS (102 ± 60%), TCDW (72 ± 35%) and SCDW (112 ± 41%), supporting the increased accuracy of this volumetric technique. Furthermore, a correlation analysis of the CRS with the different histopathological gradings showed correlation coefficients for the tibia of 0.70, 0.69 and 0.56 for the correlation with CDS, TCDW and SCDW, respectively (Fig. S22). Predictably, the least surface-sensitive SCDW score showed the poorest correlation whereas the more surface-sensitive CDS and TCDW reached a moderate correlation. For the femur, correlation coefficients were substantially lower with 0.26, 0.30 and 0.13 for the CDS, TCDW and SCDW, respectively (Fig. S23), which can be attributed to the decreased levels of cartilage degeneration, thereby decreasing the signal-to-noise ratio.

### DEX treatment leads to minor alleviation of synovitis

**Fig. 3:**
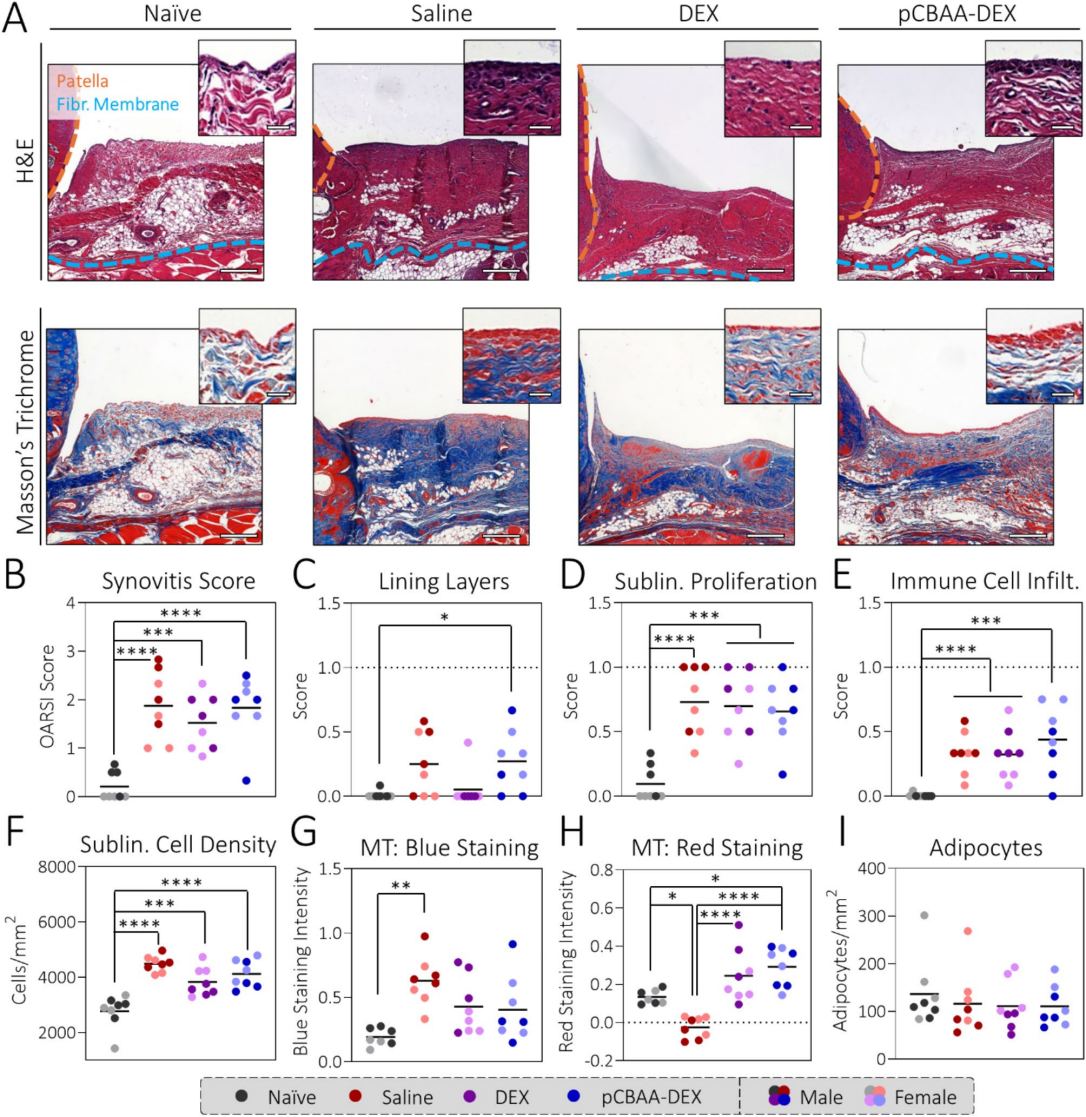
DEX treatment leads to a mild reduction of synovitis levels: (**A**) Representative histology sections of the synovia of the four study groups. Note that the synovium is bordered by the patella (orange) and fibrous membrane (blue). Scale bars: 250 μm, 25 μm (insert). (**B**) The OARSI synovitis score shows no therapeutic effect for either of the treatments. Upon examination of the three components of the synovitis score (**C**–**E**), fewer synovial lining cell layers were found for the DEX group. Quantitative analysis of the sublining cell density (**F**) revealed a minor improvement for both DEX and pCBAA-DEX compared to saline. Quantification of the color intensities in the Masson’s trichrome (MT) staining, revealed decreased blue staining (**G**) indicative of collagen density, but increased red staining (**H**) for DEX and pCBAA-DEX compared to saline. The change in red staining might be a sign of increased collagen remodeling. (**I**) The adipocyte cell density was slightly decreased for all collagenase-injected groups. *N* = 8. See Figures S4-11 for the full histology dataset. Note that all data comparisons without asterisks were statistically non-significant.

Synovitis levels were analyzed on a histological level by histopathological gradings and semi-automated image analysis (Fig. 3A). The OARSI synovitis score^38^ revealed similarly increased levels of synovial inflammation for the saline (1.88 ± 0.71), DEX (1.52 ± 0.56) and pCBAA-DEX (1.83 ± 0.67) groups compared to the naïve animals (0.21 ± 0.29, Fig. 3B). When taking a closer look at the three factors considered in the synovitis score, i.e. the number of synovial lining layers, the levels of cellular proliferation in the sublining and the infiltration of immune cells, we observed that treatment with DEX led to a decrease in number of lining layers to similar levels as in the naïve animals, which was not observed in the saline and pCBAA-DEX groups (Fig. 3C). As this effect seemed strictly limited to the lining layers but no differences were observed regarding sublining proliferation or infiltration of immune cells (Fig. 3D, E), the overall synovitis score was only marginally lower for DEX compared to the saline and pCBAA-DEX groups. Quantitative analysis of the synovial cell density via image analysis did, however, indicate differences between the treatment groups as both DEX (3831 ± 514 cells/mm) and pCBAA-DEX (4125 ± 495 cells/mm) showed slightly lower cell densities than the saline group (4486 ± 288 cells/mm, Fig. 3F). This shows the increased sensitivity of image analysis compared to categorized histopathological gradings. Moreover, we quantified the color intensities in the Masson’s trichrome stainings. For the collagens stained in blue, the two treatment groups were again located between the saline and naïve animals indicating a partial reduction in fibrosis levels (Fig. 3G). For the red color, the staining intensity for the saline group was decreased compared to the naïve animals whereas the ones for the DEX and pCBAA-DEX groups was increased (Fig. 3H). Typically, the red stain is associated with cytoplasm, muscle and keratin none of which are plausible candidates to explain the observed staining patterns^39^. This could potentially be linked to collagen remodeling as several reports have reported an increased red stain in regions with denaturated collagens, for instance in burn wounds^40,41^ or patients with cutaneous asthenia^42^. Finally, the number of adipocytes was generally similar across all conditions with a slight decrease upon collagenase injection that follows existing reports of lipodystrophy in OA (Fig. 3I)^43–45^. While the exact role of intraarticular fat tissues in OA is yet to be fully elucidated, they are thought to play a crucial role in maintaining a healthy joint phenotype^46^. Conversely, their loss due to fibrotic remodeling or adipocyte death has been associated with an increased immune cell presence^45^, elevated levels of pro-inflammatory cytokines^47^ and increased cartilage degeneration^48^.

### pCBAA-DEX shows increased retention in cartilage compared to other joint tissues

**Fig. 4:**
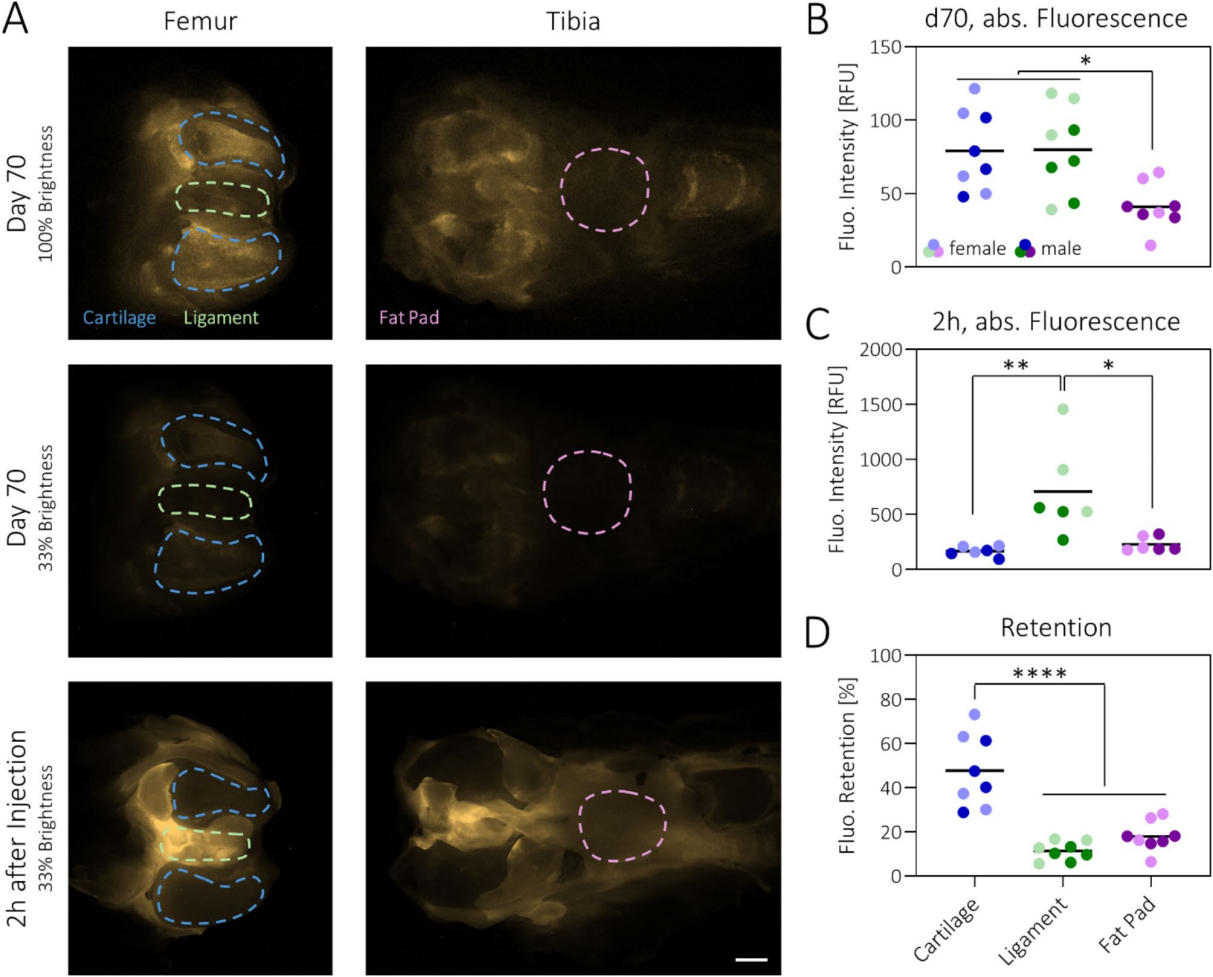
pCBAA-DEX achieves 50% cartilage retention over 70 days: (**A**) Fluorescence micrographs of representative femur and tibia samples imaged after 70 days in vivo or 2 h after polymer injection. The dotted lines signify the quantified areas for the analysis. Scale bar: 2 mm. (**B**) Quantification after 70 days indicates increased polymer amounts in cartilage and ligaments compared to the fat pad. (**C**) Immediately after injection, there is substantially more polymer in the ligaments than in cartilage and fat pad. (**D**) Overall, pCBAA-DEX is retained substantially better in cartilage than in ligaments and fat pad. *N* = 8. Note that all data comparisons without asterisks were statistically non-significant.

To evaluate the biological fate of the pCBAA-DEX polymer, it was made fluorescent by including a rhodamine B comonomer. Using fluorescence stereomicroscopy, the polymer distribution in different joint tissues was visualized on day 70 of the study (Fig. 4A). Comparison of fluorescence intensities revealed similar values in femoral cartilage and ligaments that were increased compared to the fat pad (Fig. 4B). While this suggests comparable total polymer amounts in cartilage and ligaments, the intra-tissue concentration was most certainly substantially higher in the cartilage, as it is a lot thinner than the ligaments and the light detected by the microscope was emitted from a much smaller volume. To estimate the retention levels throughout our study, fluorescence levels on day 70 were compared to images taken only 2 h after intra-articular injection into rat cadaver knees (Fig. 4A). At this timepoint, the fluorescence intensity was greatly increased in the ligaments compared to both cartilage and fat pad (Fig. 4C). Comparing the two timepoints, pCBAA-DEX retention levels of 48 ± 16%, 11 ± 4% and 18 ± 7% were calculated for cartilage, ligaments and fat pad, respectively (Fig. 4D).

To investigate whether any of the released polymer had accumulated elsewhere, liver, lung, heart, kidney and spleen were imaged analogously. None of the organs showed any increase in fluorescence compared to control animals (Figure S23).

### No effect of treatments on subchondral and trabecular bone

**Fig. 5:**
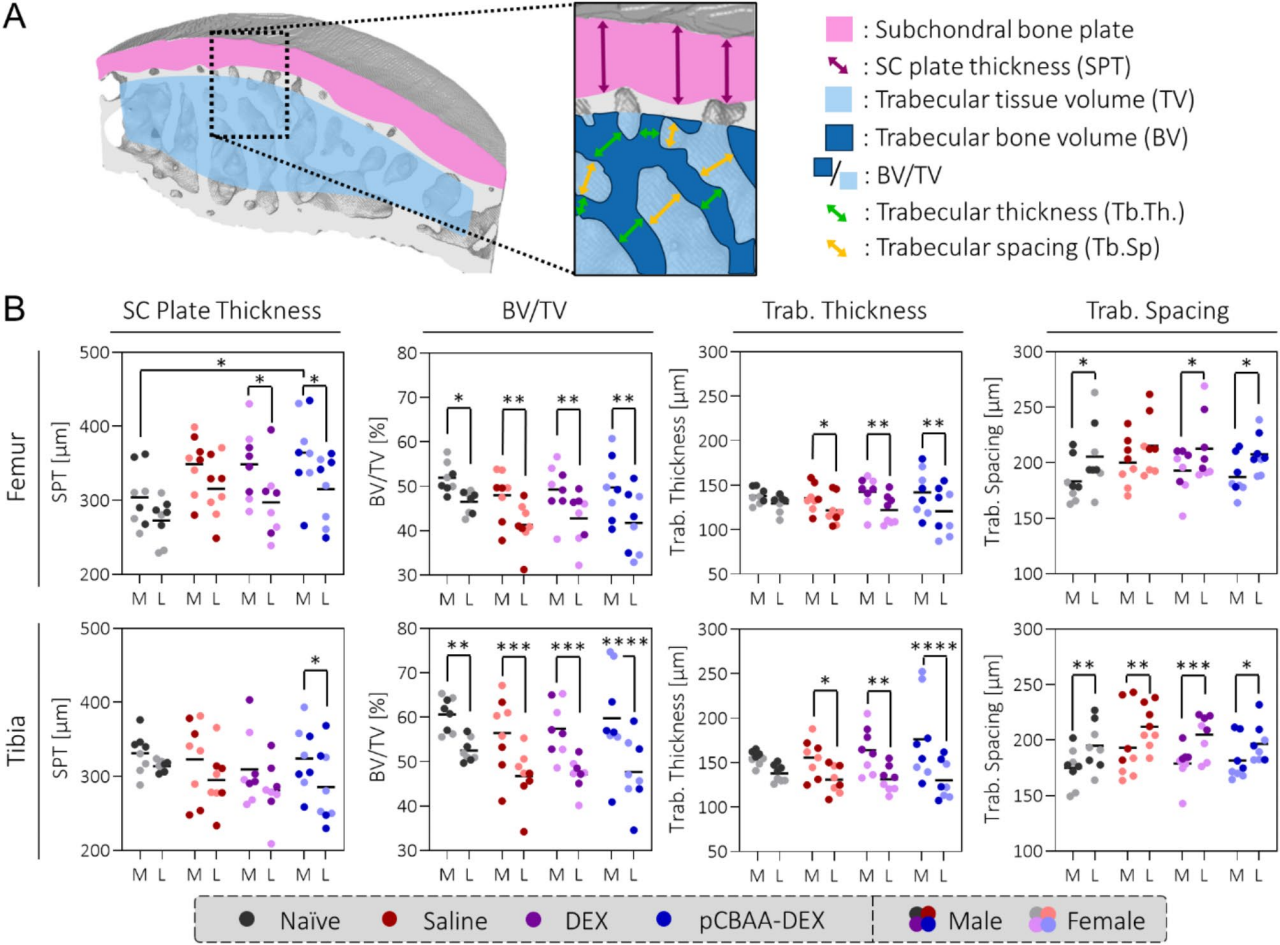
DEX and pCBAA-DEX do not influence bone resorption triggered by collagenase injection: (**A**) Schematic illustration of the bone metrics extracted from the computed tomography scans. Illustration reproduced with permission from *Weber et al.*^28^. (**B**) Apart from the femoral subchondral bone plate thickness, there was a general trend towards bone resorption upon injection of collagenase for all of the investigated bone metrics. Neither DEX nor pCBAA-DEX, however, led to any changes in comparison to the saline group. Bone resorption was generally increased in the tibia compared to the femur. M: medial, L: lateral, *N* = 8. Note that all data comparisons without asterisks were statistically non-significant.

Following previous reports of bone resorption in the CIOA model^28,31^ and the fact that DEX exposure is known to be a risk factor in that regard^49^, we performed subchondral and trabecular bone analysis on the computed tomography scans (Fig. 5). Our dataset also showed a general trend towards bone resorption upon collagenase injection, however, neither DEX nor pCBAA-DEX led to any changes in comparison to the saline group.

### No effect on gait asymmetry and weight

**Fig. 6:**
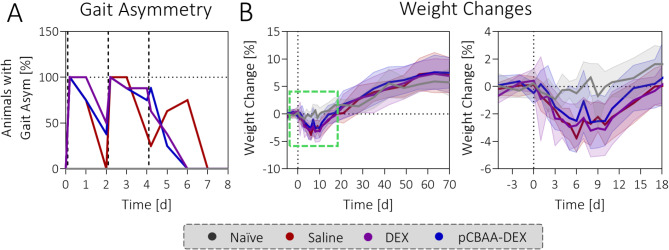
DEX and pCBAA-DEX have no effect on gait asymmetry and weight loss: (**A**) Visually observed gait asymmetry in the animals was no longer detected beyond day 7 of the study. There are no statistically significant differences between saline, DEX and pCBAA-DEX groups. The vertical lines represent the timepoints for intraarticular injection (day 0 & 2: collagenase, day 4: treatments). (**B**) All treatment groups show a similar decrease in weight of 2–4% between days 6 and 10 of the study. Beyond day 10, the animals start to regain weight and follow a similar trajectory as the naïve animals. The solid line represents the average value, the area the standard deviation.

Generally, all our treatments were well tolerated by the animals. Similar to previous observations^28^, the discomfort caused by the collagenase injection was transient and there were no sustained visual changes in gait after day 6 (Fig. 6A). Moreover, the observed weight loss also followed a similar trajectory as reported previously, with an average weight loss of around 4% between days 6 till 10 beyond which the animals started to gain weight again (Fig. 6B). Neither DEX nor pCBAA-DEX had any noticeable effect on the changes in gait or animal weight compared to the saline group.

### Investigation of the sex differences

Sex is one of the main risk factors in human OA^50^, and we also observed some sex-related differences in our dataset (Fig. S24). For the cartilage metrics, the females were found to have increased levels of cartilage degeneration compared to the males. This trend was, however, only present for the histopathological gradings but not the CRS, where no sex difference was observed. For the synovium, the trend went in the opposite direction, with the males generally showing a more severe phenotype than the females. Compared to the cartilage, this trend was less pronounced, as none of the differences were statistically significant.

## Discussion

The main goal of this study was to assess the therapeutic potential of the slow-release pCBAA-DEX conjugate in a more clinically relevant model compared to previous in vitro experiments. The conclusion is inevitably that our results do not indicate any therapeutic activity in this setting. While there might have been a minor improvement for the synovium, the pCBAA-DEX group showed an increase in cartilage degeneration compared to the saline-injected animals. Furthermore, for the synovium, the pCBAA-DEX group was still outperformed by DEX which was able to prevent the increase in synovial lining layers. Given the extremely short-lived effect of DEX in the joint capsule, this implies that the synovial phenotype that we saw on day 70 was mostly determined during the first few days after collagenase injection. While the pCBAA-DEX conjugate was releasing DEX into the joint over a more extended period, it seems that the intra-articular concentrations at any given time were either too low to have an effect or too late, as many of the remodeling processes had already progressed too far to be reversed within the timeframe of this study. Based on the available literature, the former is probably the more likely of the two explanations, as most studies reporting therapeutic effects with highly targeted DEX-based solutions typically injected substantially higher amounts and/or repeated the injections multiple times throughout the study^9,10,51–53^. In addition, the Avidin-DEX construct developed by *Bajpayee et al*. combines fast- and slow-release chemistries^10,11^ that could have also helped to achieve a better therapeutic effect in this PTOA-mimicking animal model.

While increasing the DEX dose and/or switching to a treatment scheme with repeated administrations would certainly help to improve the effectiveness of our conjugate, it might still not be enough to trigger a robust therapeutic effect, and this approach also contradicts our goal of reducing the required DEX dose by delivering it in a targeted manner. Alternatively, DEX availability could also be increased by improving the targeting as most of the conjugate in our study was rapidly absorbed by soft tissues other than cartilage. As OA is increasingly understood as a whole joint disease^54^, uptake into tissues other than cartilage could also elicit a therapeutic effect. However, as the concentration of GAGs and thus the overall negative charge is substantially lower in ligaments and fat pad compared to cartilage^55–57^, the polymer retention in these tissues was substantially lower. Compared to isolated, cleaned rat tibias and femurs without surrounding soft tissues that were submerged in pCBAA-DEX solutions at an equivalent dose, the cartilage fluorescence after intra-articular injection was around 85% lower, thus indicating substantial uptake into non-cartilaginous tissues with poor polymer retention (Figure S25).

To further improve the retention of our pCBAA-DEX conjugate within the joint cavity, there are several approaches that could be pursued: Firstly, a less specific targeting mechanism that does not require the presence of GAGs in the tissue might be beneficial to increase the whole joint retention. In a previous study, we had investigated a pCBAA-dopamine copolymer that was discarded at the time due to decreased penetration into cartilage but might be more suitable for other soft tissues^16^. A higher degree of surface localization might also improve its functionality as a boundary lubricant, which, based on the cartilage results in this study, was clearly not effective enough with the current formulation. Another approach would be to increase the molecular weight of the conjugate by incorporating DEX into bulkier bottlebrush polymers^23^ or even macroscopic (granular) hydrogels. The latter approach is well established in the literature, with systems based on HA^52,58^, chitosan^59^ or zwitterionic sulfobetaines^60^ being evaluated in OA preclinical animal studies. While the use of macroscopic (granular) hydrogels has the potential to greatly improve the retention kinetics, the lubrication properties would be very different, as they would not serve as a boundary lubricant at the cartilage surface. In either case, a future study investigating one or several improved targeting mechanisms should ideally include additional timepoints to better understand the homing and drug release kinetics in vivo to optimally time its administration and dosing with respect to the development of the OA-like phenotype in the animals.

Despite the discussed limitations, none of them can explain why a greater degree of cartilage degeneration was observed for the pCBAA-DEX group compared to the saline-injected animals. While the anti-inflammatory and lubricating properties of our conjugate clearly did not fully translate from the in vitro to the in vivo setting, we had not expected a harmful effect. As the cartilage degeneration levels in the DEX group are indistinguishable from saline-injected animals, we assume that the observed increase can be entirely attributed to the pCBAA polymer chemistry. The most likely explanation is that the positive charge of the pCBAA polymer led to unfavorable interactions with other biomolecules, thereby accelerating the rate of cartilage degeneration. It is well known that polycationic agents such as poly-ethylenimine or poly-L-lysine can trigger cell membrane instability and DNA complexation, both of which resulting in detrimental effects on cell health^61^. Moreover, the association of cationic agents with anionic GAGs is also known to influence the mechanical properties of cartilage tissue which may biomechanically trigger a change in chondrocyte metabolism^62–64^. While previous studies on pCBAA polymers have found both a dose-dependent decrease in metabolic activity and decreased stress-relaxation rates in bovine cartilage explants, these experiments required substantially higher intra-cartilage concentrations than are expected in this study. Nevertheless, there are substantial differences between cartilage tissues of different species that may compromise comparability^65^. Another possible explanation is that anionic ECM fragments released by collagenase digestion or cell-mediated ECM degradation were captured by the polymers and acted as damage-associated molecular patterns, thereby triggering additional inflammation and cartilage degeneration^66,67^. Finally, a recent study also found that upon intra-articular injection of an even higher dose of linear pCBAA polymers, a substantial increase in immune cell infiltration into the synovium was observed in addition to increased cartilage degeneration^68^. Though no such observation was made for the synovia in our study, an immunological contribution cannot be completely ruled out. Ultimately, further experiments that also include different doses and additional timepoints will be necessary to fully understand the causes and sequence of events triggered by intra-articularly injected pCBAA-based materials in vivo.

## Conclusion

While the results did not support the translational potential of our pCBAA-DEX conjugate, there are still many things that can be learned from this study. Most notably, the in vitro findings clearly did not translate into the more complex CIOA animal model, which questions the in vitro models that were used. In that respect, simply focusing on the cartilage was not enough, as based on tissue volumes and the relative surface areas within the joint cavity, intra-articularly injected therapeutics are far more likely to end up in other soft tissues than in the cartilage. While several multi-tissue joint-on-a-chip models do exist that could provide a more relevant environment for such studies, they are most commonly based on microfluidic setups that fail to fully recapitulate the physiological organization of the different joint tissues^69,70^. Furthermore, the technical difficulty in setting up these models also represents a major limitation towards their wide-spread use, and we believe co-culture models of tissue explants to be a good compromise. To study intra-articular penetration and retention, we strongly recommend the use of cadavers, as this allows for the most representative evaluation of kinetic parameters and tissue distribution of the injected compound before going in vivo.

Despite the failure of our pCBAA-DEX conjugate, the in vitro lubrication and drug delivery properties of the pCBAA carrier itself remain valid, however, translating them to a more clinically relevant setting might require a substantial redesign of our system, especially in light of the observed increase in cartilage degeneration for pCBAA-DEX. More concretely, separating the lubricating and drug delivery functionalities into two separate therapeutics could prove greatly beneficial, as their requirements do not always align. While a boundary lubricant is most effective when bound specifically to the cartilage surface, an anti-inflammatory drug such as DEX is most effective if delivered not only to the cartilage but also to other involved tissues such as the synovium and the fat pad. To achieve satisfactory retention kinetics of the drug within the joint cavity, linear, soluble polymers have some inherent drawbacks, and a substantial loss of the initial dose shortly after injection seems inevitable. Macroscopic carriers, on the other hand, can evade clearance through the capillary fenestrations of the synovium more effectively. Therefore, we could envision a combination product of granular pCBAA-DEX hydrogels and high molecular weight, surface-reactive pCBAA to be the most effective at providing sustained DEX release as well as boundary lubrication to the joint.

Regardless of which structure and chemical basis the next generation of OA drug candidates will have, the methodology that was pursued in this study has provided reliable data for assessing the therapeutic potential of any given therapeutic. Though conventional histopathological gradings were included for the evaluation of both cartilage and synovium tissues, they were accompanied by quantitative methods that do not rely on the subjective impression of a human grader, thereby providing a more accurate and reproducible readout. We hope that with this study, we can inspire researchers to rethink their methods not just with respect to their in vitro experiments but also for the evaluation of animal experiments, to ultimately improve the chances for clinical translation of therapeutic candidates in the future.

## Electronic supplementary material

Below is the link to the electronic supplementary material.

Supplementary Material 1

## Data Availability

The datasets generated and analysed during the current study are available in the ETH Zurich Research Collection under the DOI:10.3929/ethz-b-000691443.
