# Supplementary material for: Zwitterionic poly-carboxybetaine-dexamethasone conjugates do not alleviate cartilage degeneration and synovitis in the collagenase-induced osteoarthritis model in rats
Source: Sci Rep. 2025 Jul 1;15:20501. doi: 10.1038/s41598-025-93247-3 (PMC12215361; doi:10.1038/s41598-025-93247-3)
Supplement: Supplementary file 1 — Supplementary Material 1 [file 41598_2025_93247_MOESM1_ESM.pdf]

Supplementary Information

Zwitterionic Poly-Carboxybetaine-Dexamethasone Conjugates Do Not Alleviate Cartilage Degeneration and Synovitis in the Collagenase-Induced Osteoarthritis Model in Rats

Patrick Weber[1], Maryam Asadikorayem[1], Shipin Zhang[1], David Fercher[1], Kajetana Bevc[1], Sami Kauppinen[2], Tuomas Frondelius[2], Tianqi Zhang[2], Marina Fonti[1], Gonçalo Barreto[3], Mikko A.J. Finnilä[2,4], Marcy Zenobi-Wong[1*]

[1]Tissue Engineering + Biofabrication Laboratory, Department of Health Sciences and Technology, ETH Zürich, Otto-Stern-Weg 7, 8093 Zürich, Switzerland

[2]Research Unit of Health Sciences and Technology, University of Oulu, Aapistie 5A, 90220, Oulu, Finland

[3]Clinicum, Faculty of Medicine, University of Helsinki and Helsinki University Hospital, Haartmaninkatu 8, 00290, Helsinki, Finland

[4]Biocenter Oulu, University of Oulu, Aapistie 5A, 90220, Oulu, Finland

***Corresponding author E-mail: marcy.zenobi@hest.ethz.ch**

Enzyme	Activity
Collagenase	290 U/mg
Caseinase	535 U/mg
Clostripain	4.70 U/mg
Trypsin	0.58 U/mg
FALGPA	0.14 U/mg

Table S1 – Collagenase Enzymatic Activities: Low-activity batch of collagenase with the following enzymatic activities as measured by Stemcell Technologies. Enzymatic activity was documented using the FALGPA assay in-house prior to injection into the animals.

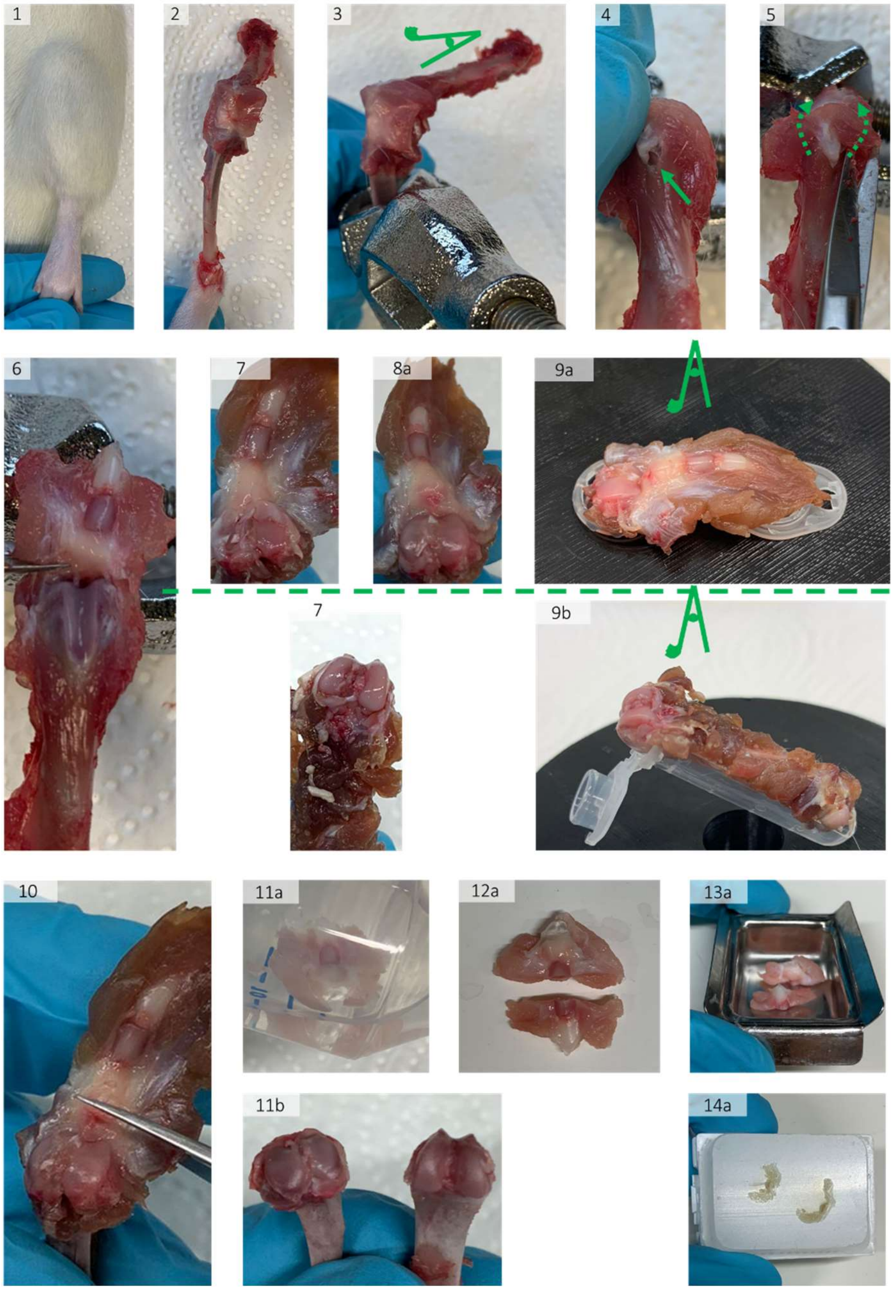

Figure S1: Step-by-step illustration of joint tissue harvest:

1) Intact rat knee joint
2) Leg removed by dislocation of hip joint. Capsule intact, all other tissues removed.
3) Paw removed and tibia clamped for fixation. Sample turned to have back view on joint capsule for step 4 (green eye).
4) Incision made proximally to the patella, above the trochlear groove.
5) Joint capsule opened by cutting around the patella.
6) Image of open joint capsule.
7) Femur and tibia separated after transsection of ACL, PCL and other connecting tissues.

8a) Menisci removed from tibia.
9a/b) Samples were imaged by fluorescence microscopy from the top. Femur was positioned at an angle to maximise the imaged surface area of the condyles.
10) Peripatellar joint capsule cut away from tibia
11a) Peripatellar joint capsule fixed and decalcified.
11b) Remove soft tissues from femur/tibia for subsequent μCT analysis.
12a) Peripatellar joint capsule halved in transverse direction through patella.
13a) Tissues paraffinized and embedded with cut plane facing downwards.
14a) Histology blocks with peripatellar joint capsule crosssection.
Image was modified from Weber et al.(1)

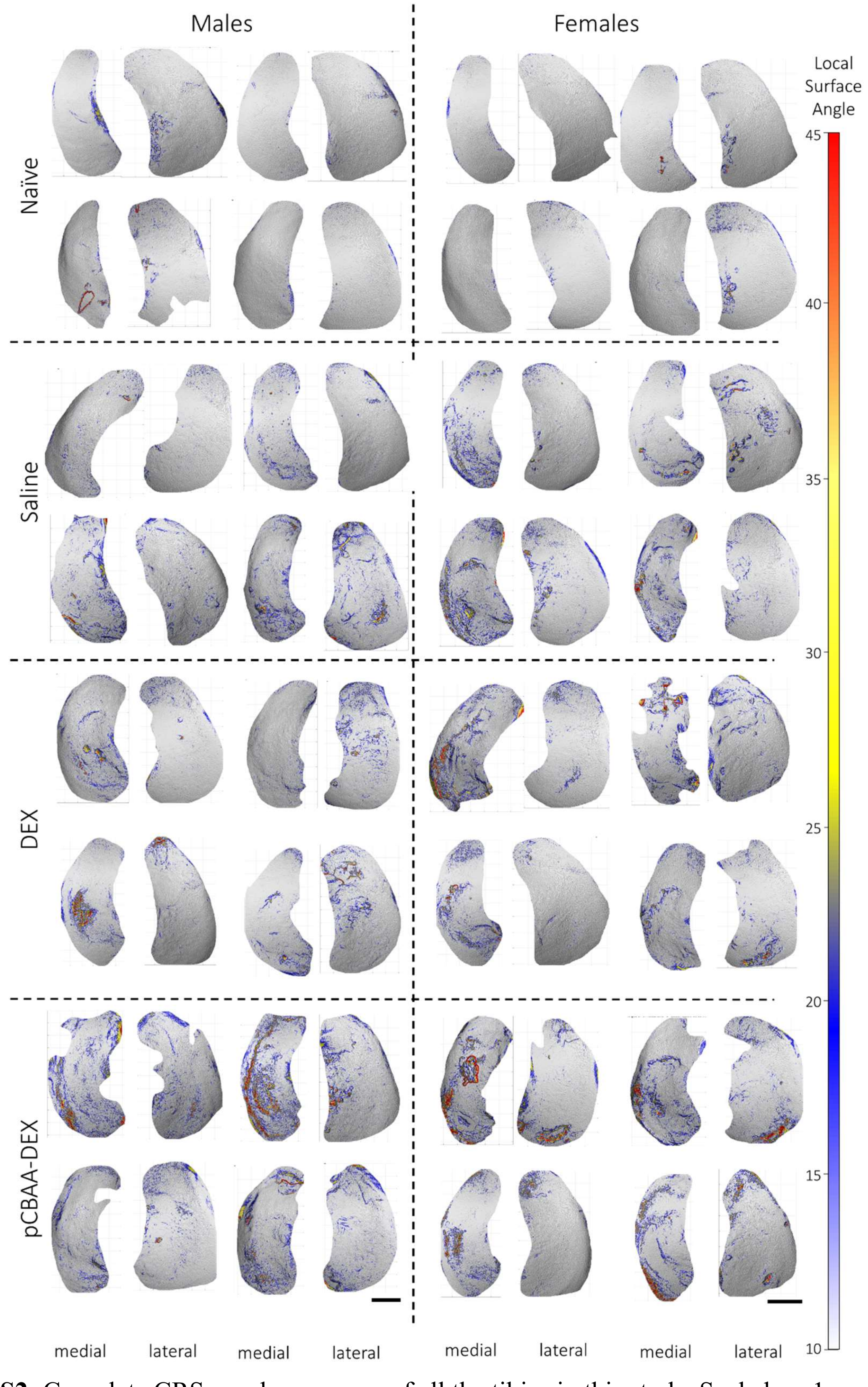

Figure S2: Complete CRS roughness maps of all the tibias in this study. Scale bar: 1 mm.

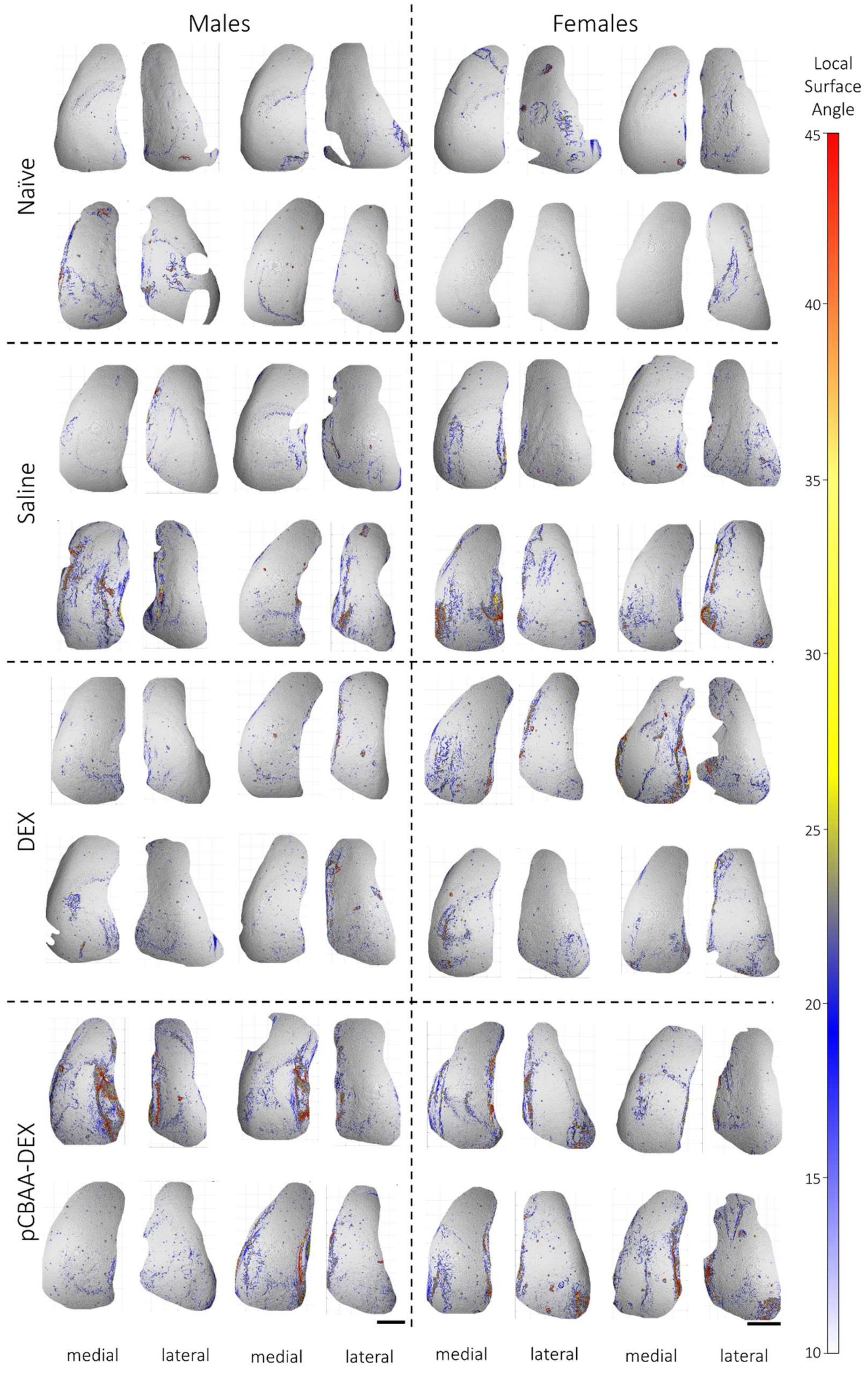

Figure S3: Complete CRS roughness maps of all the femurs in this study. Scale bar: 1 mm.

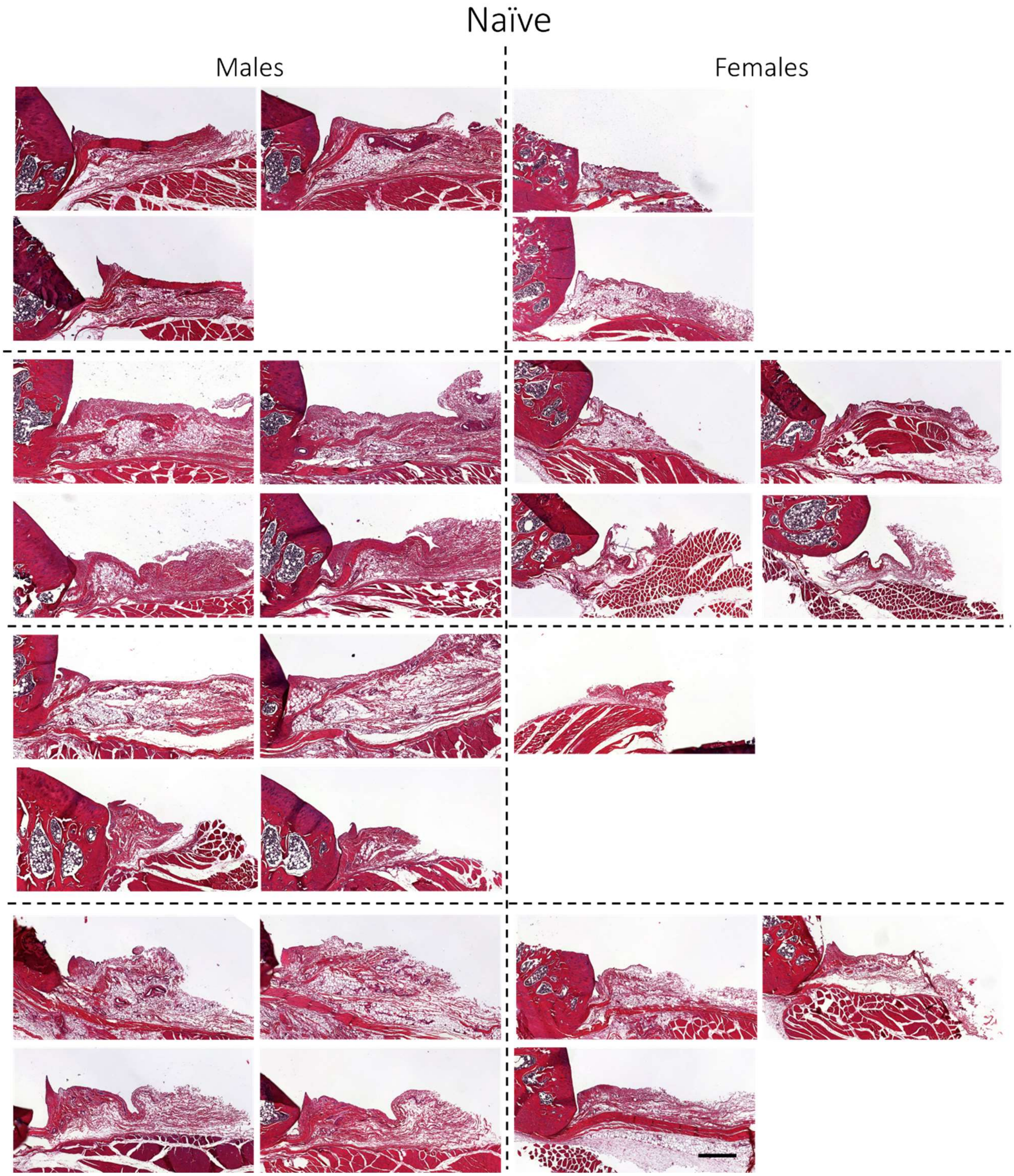

Figure S4: Complete H&E histology of all the synovia in the naïve group. There are two sections perjoint with two ROIs per section medially and laterally of the patella. Scale bar: 500 μm.

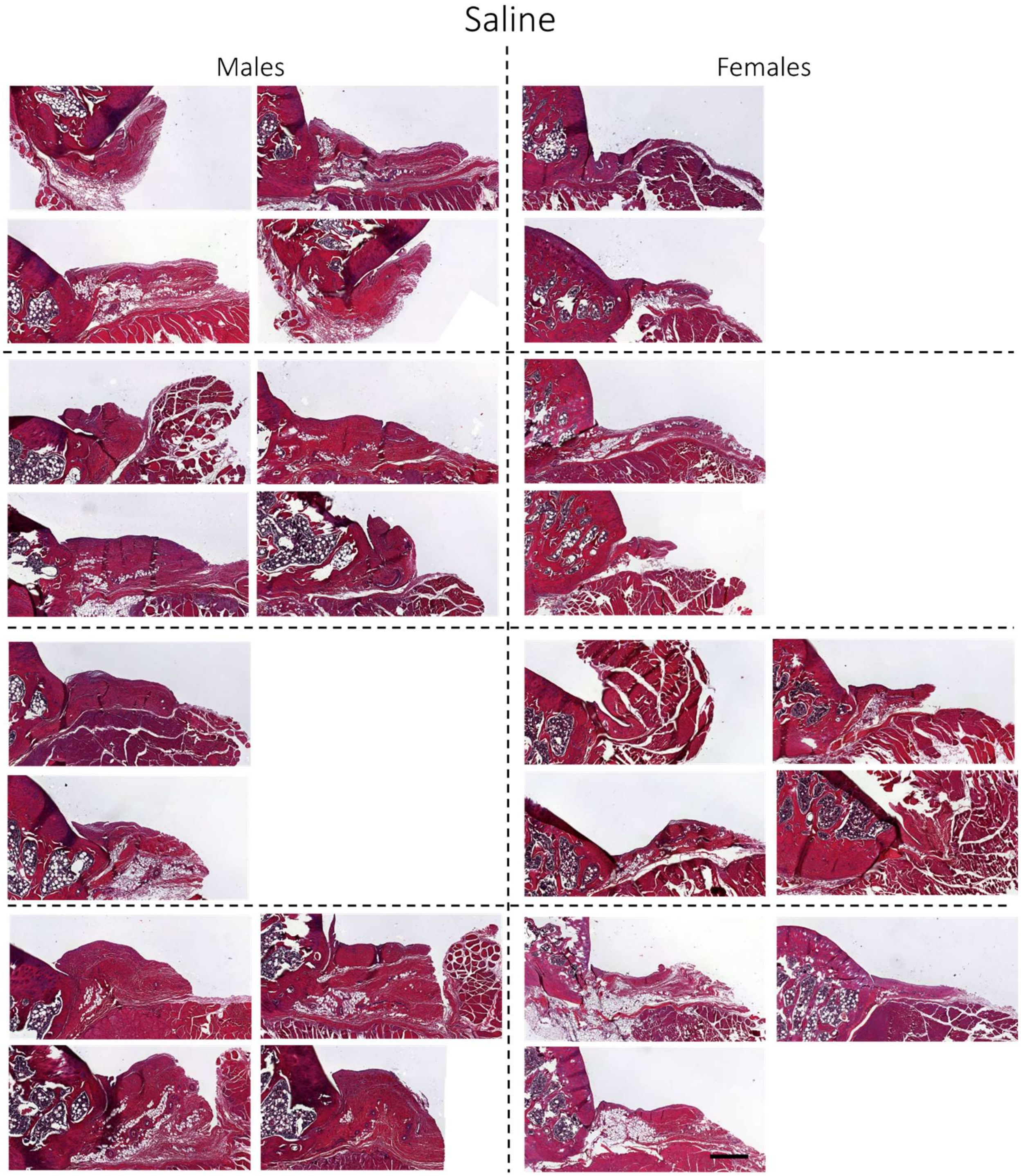

Figure S5: Complete H&E histology of all the synovia in the saline group. There are two sections perjoint with two ROIs per section medially and laterally of the patella. Scale bar: 500 µm.

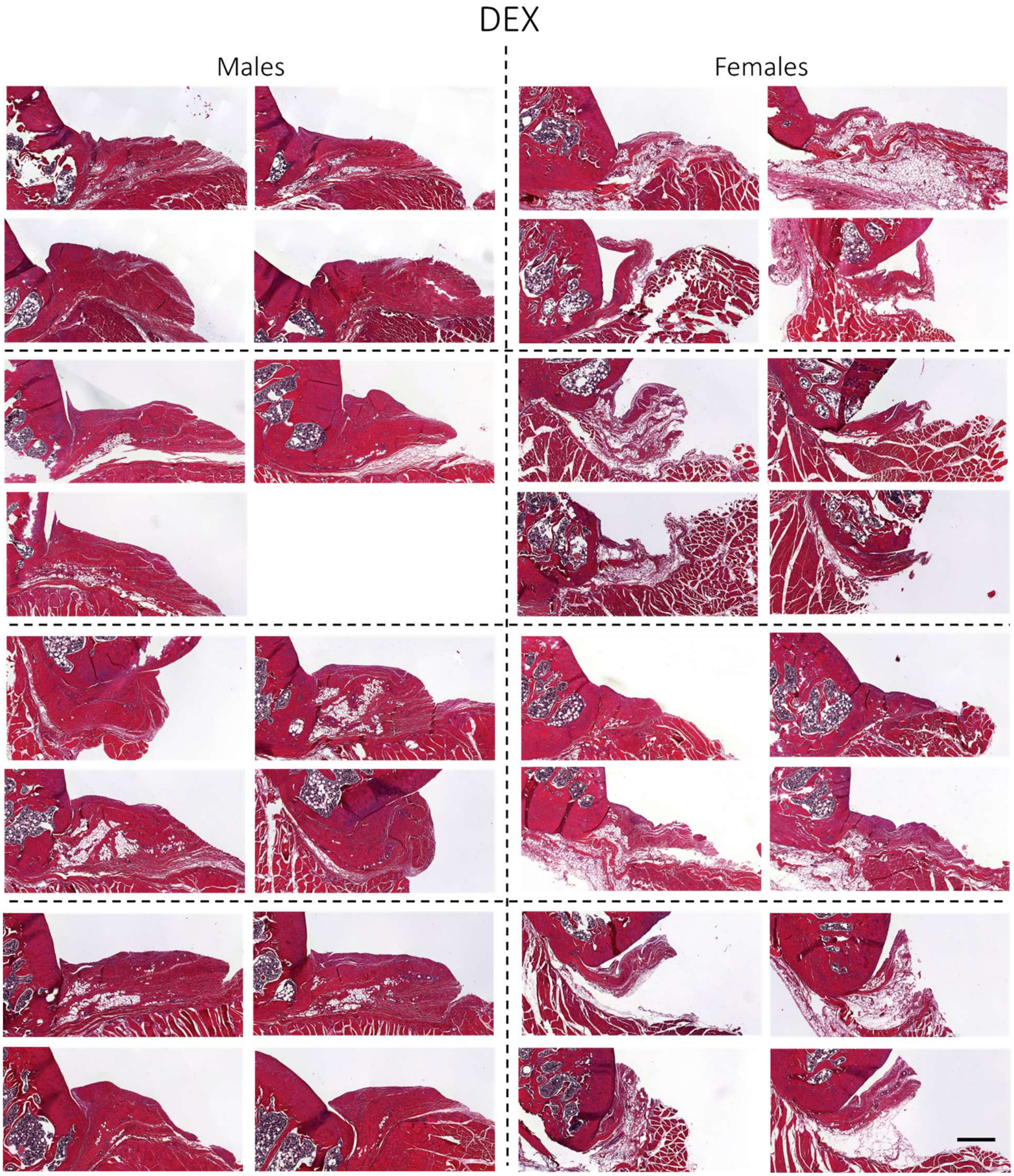

Figure S6: Complete H&E histology of all the synovia in the DEX group. There are two sections perjoint with two ROIs per section medially and laterally of the patella. Scale bar: 500 µm.

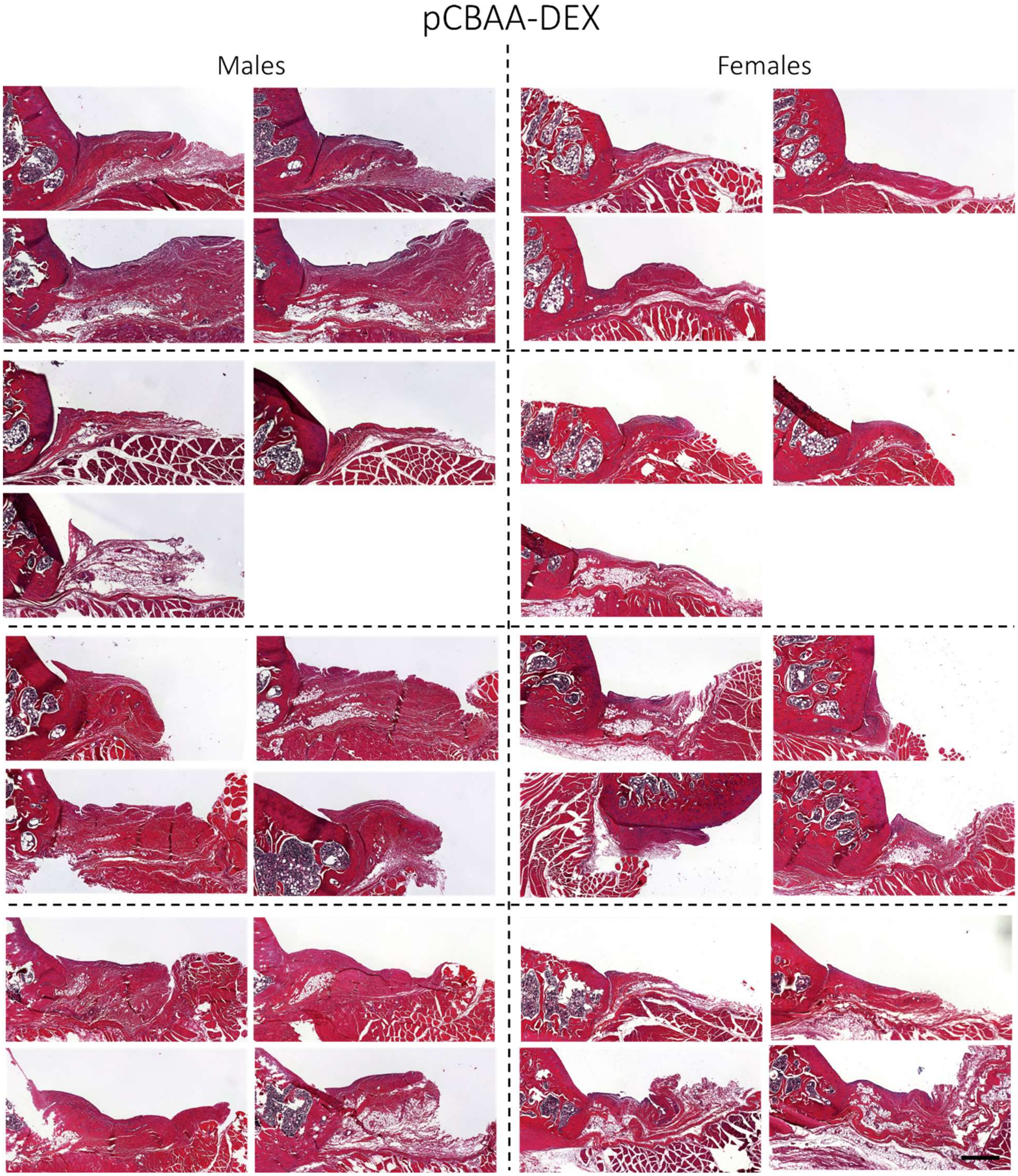

Figure S7: Complete H&E histology of all the synovia in the pCBAA-DEX group. There are two sections perjoint with two ROIs per section medially and laterally of the patella. Scale bar: 500 μm.

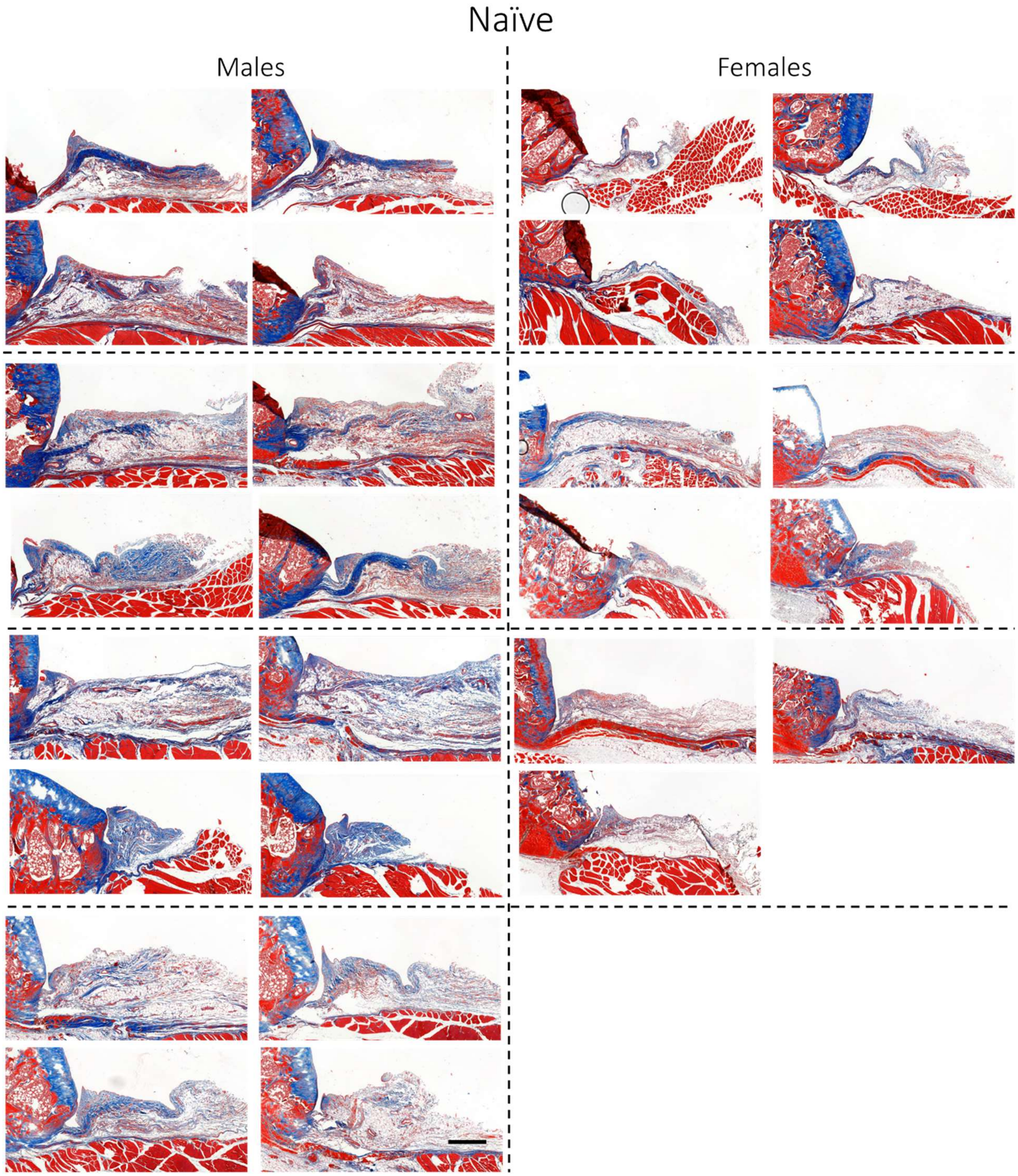

Figure S8: Complete Masson's trichrome histology of all the synovia in the naïve group. There are two sections perjoint with two ROIs per section medially and laterally of the patella. Scale bar: 500 μm.

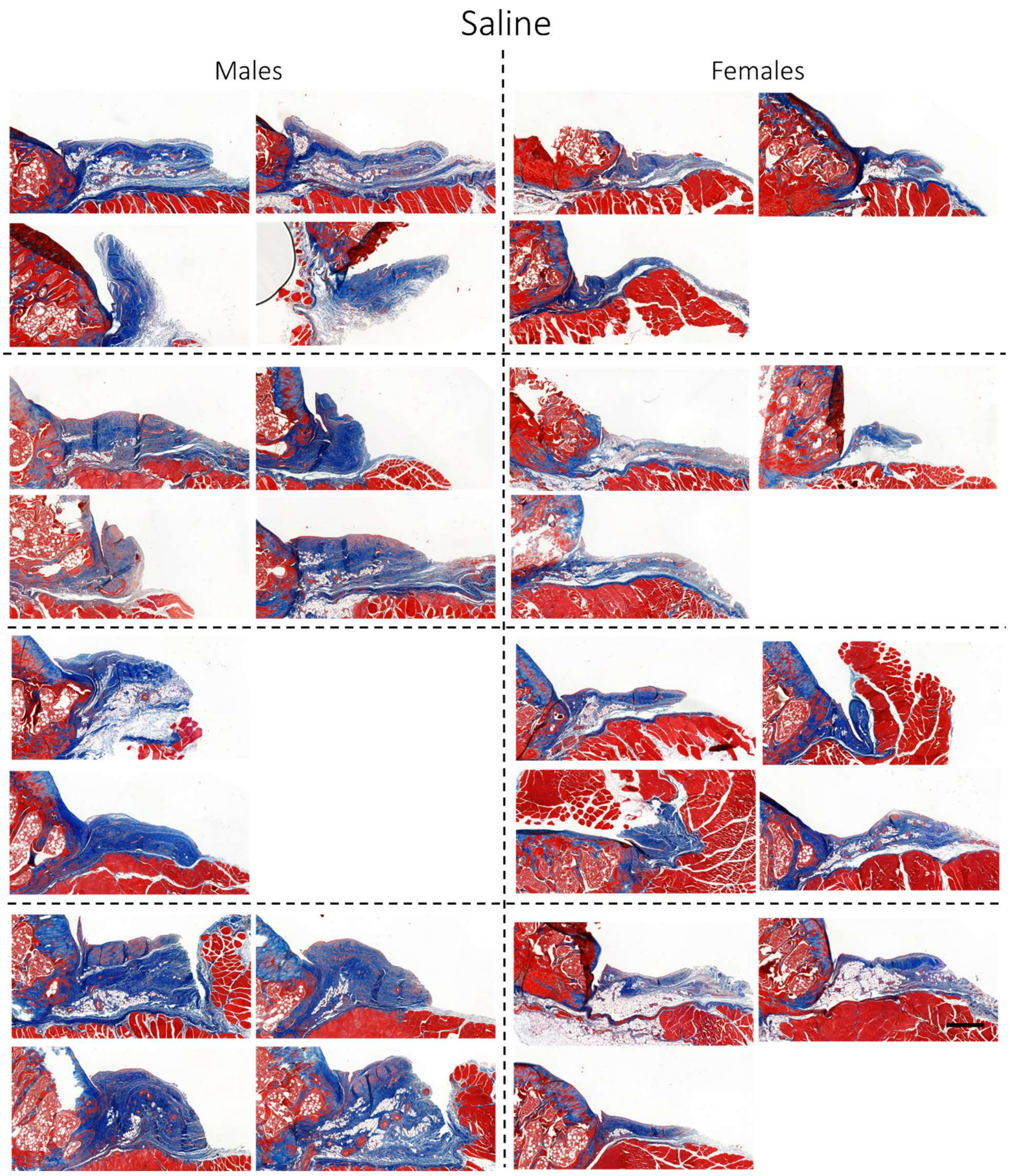

Figure S9: Complete Masson's trichrome histology of all the synovia in the saline group. There are two sections perjoint with two ROIs per section medially and laterally of the patella. Scale bar: 500 µm.

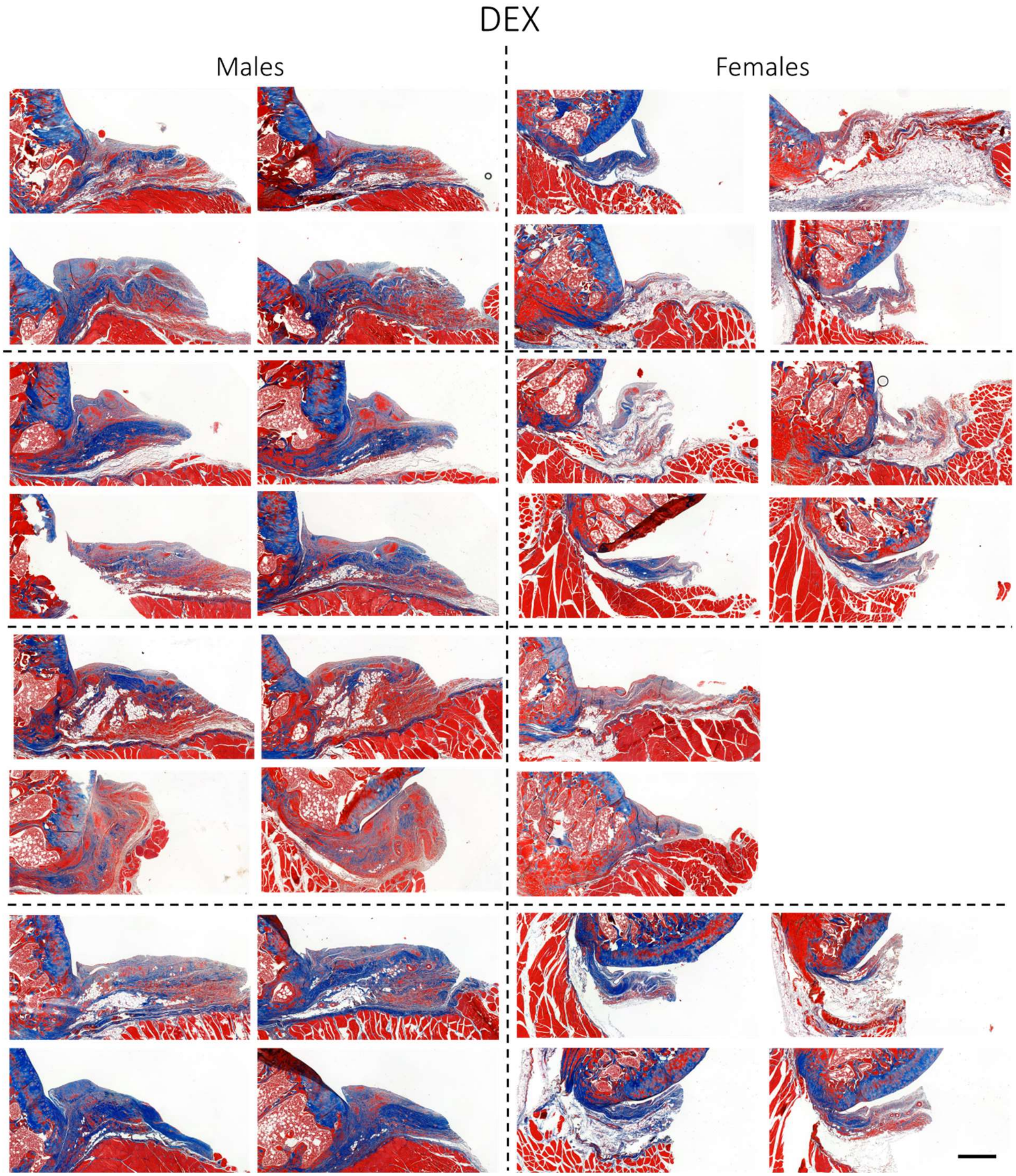

Figure S10: Complete Masson's trichrome histology of all the synovia in the DEX group. There are two sections perjoint with two ROIs per section medially and laterally of the patella. Scale bar: 500 µm.

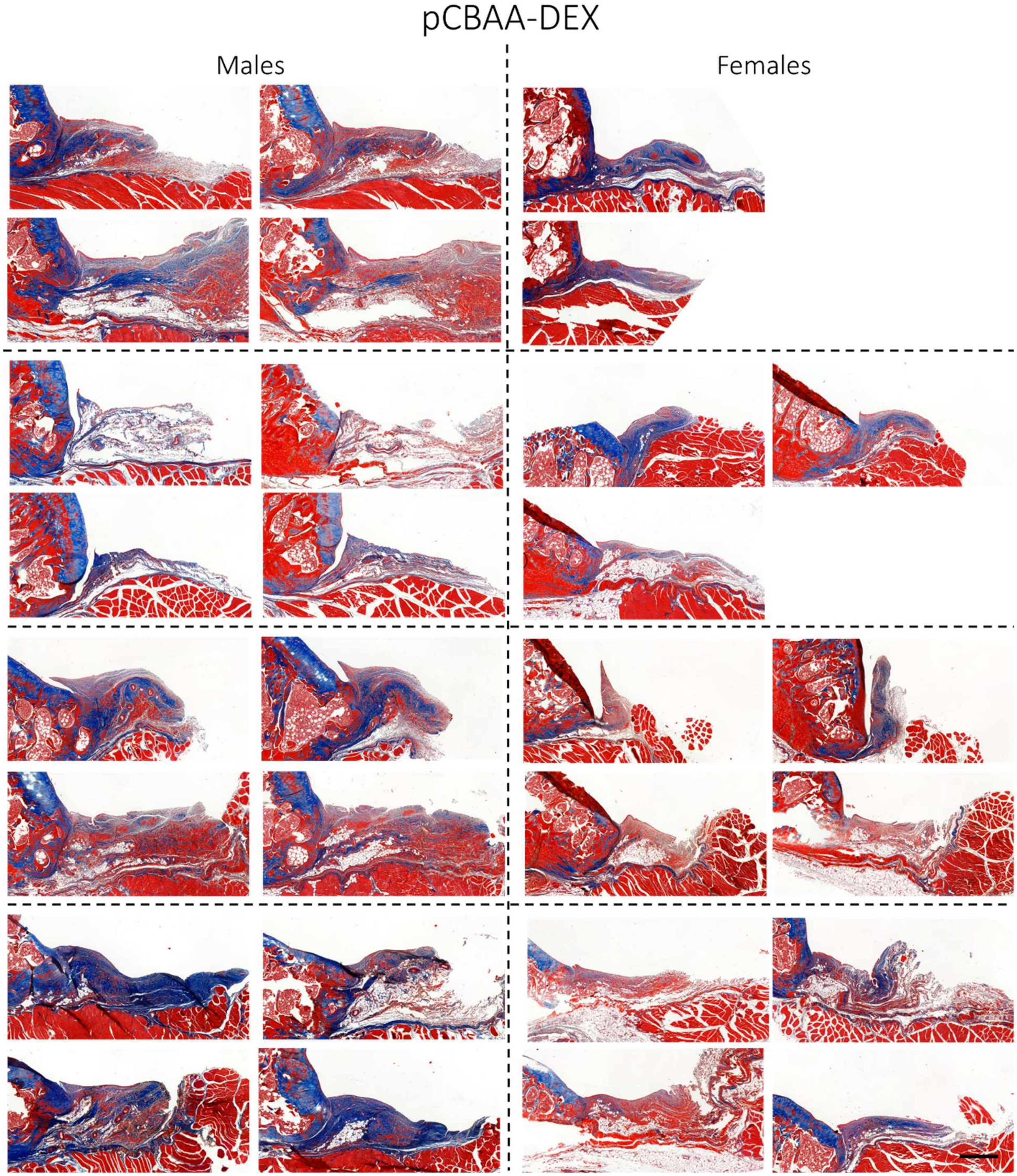

Figure S11: Complete Masson's trichrome histology of all the synovia in the pCBAA-DEX group. There are two sections perjoint with two ROIs per section medially and laterally of the patella. Scale bar: 500 μm.

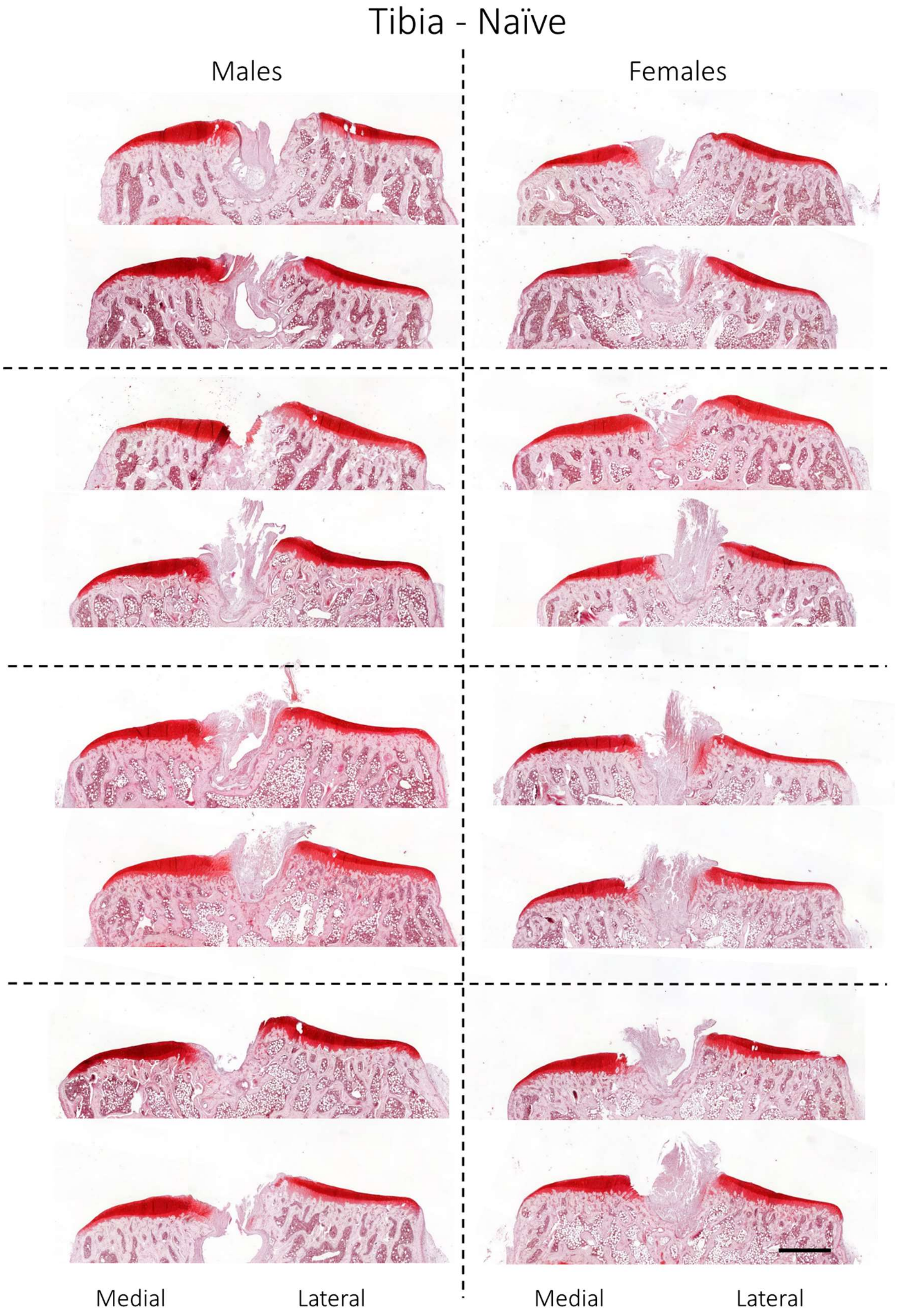

Figure S12: Complete safranin O histology of all the tibias in the naïve group. Scale bar: 1 mm.

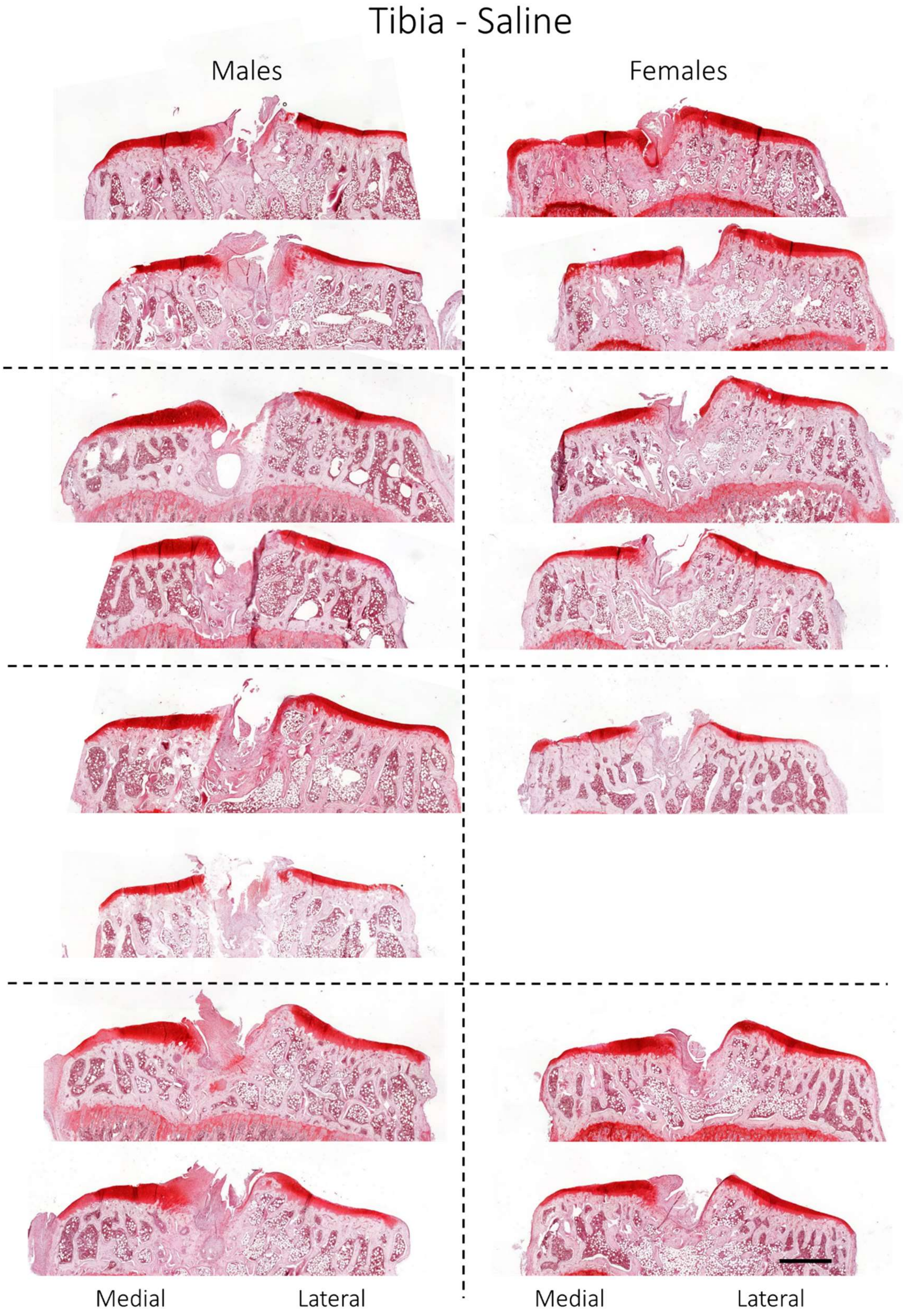

Figure S13: Complete safranin O histology of all the tibias in the saline group. Scale bar: 1 mm.

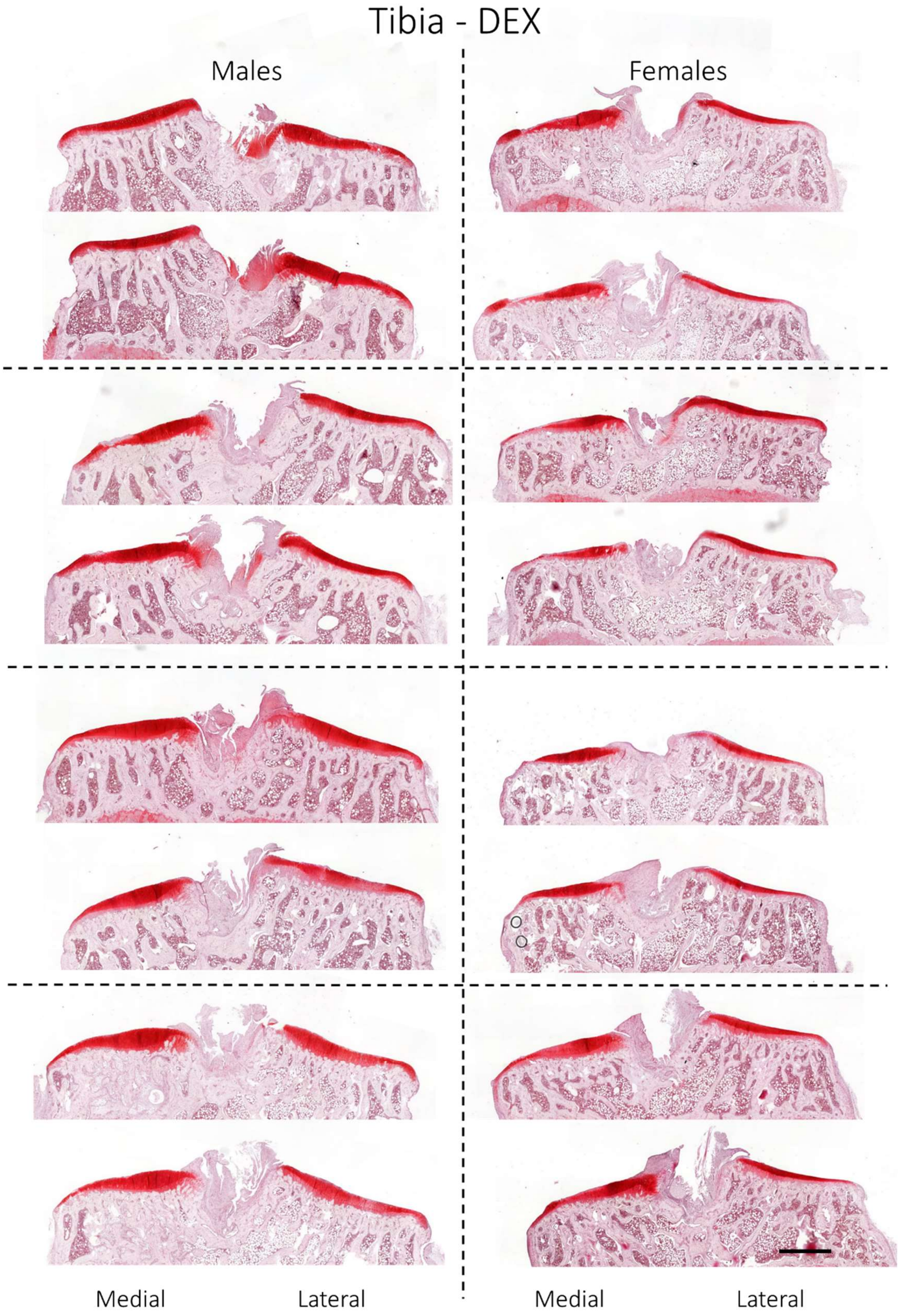

Figure S14: Complete safranin O histology of all the tibias in the DEX group. Scale bar: 1 mm.

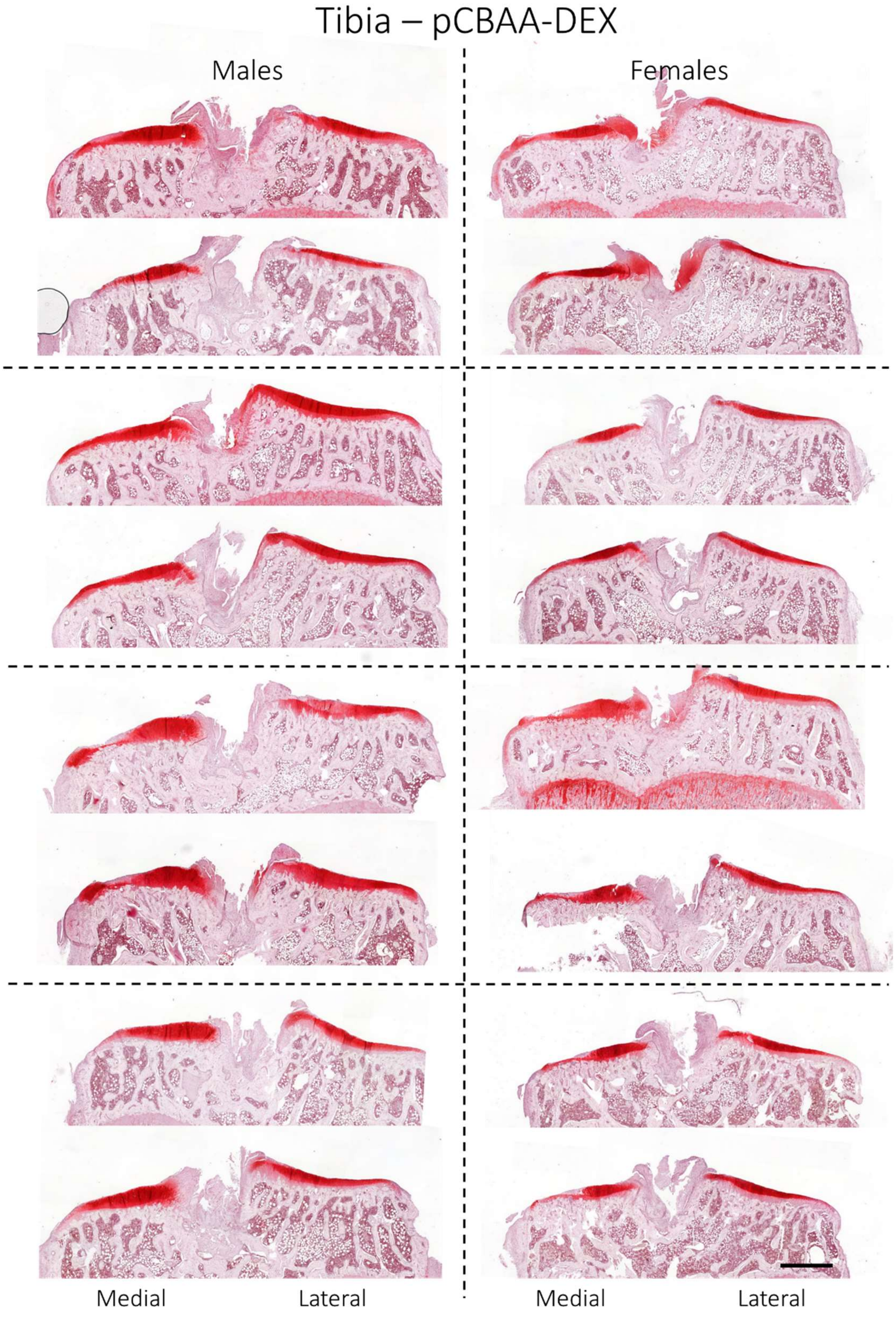

Figure S15: Complete safranin O histology of all the tibias in the pCBAA-DEX group. Scale bar: 1 mm.

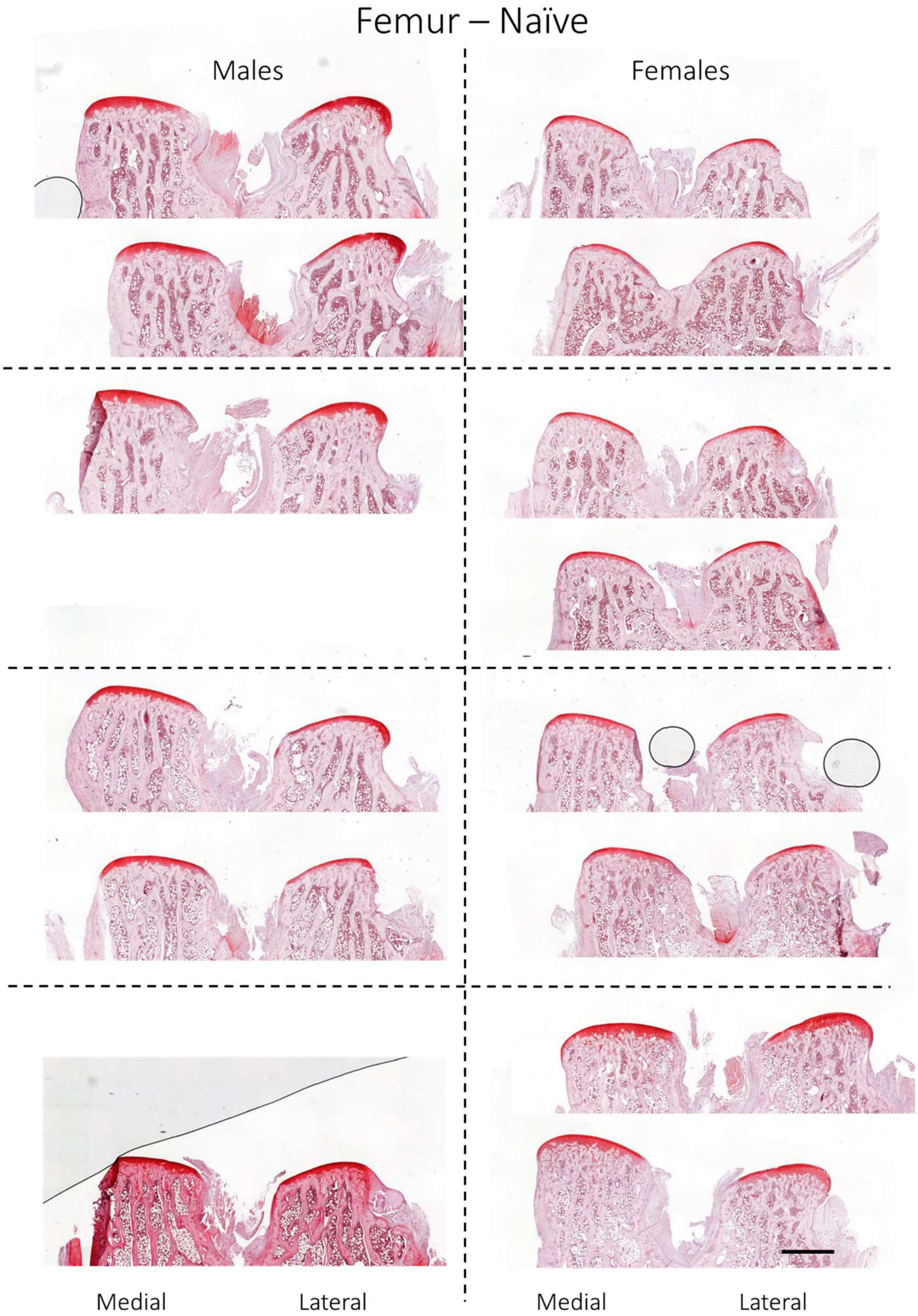

Figure S16: Complete safranin O histology of all the femurs in the naïve group. Scale bar: 1 mm.

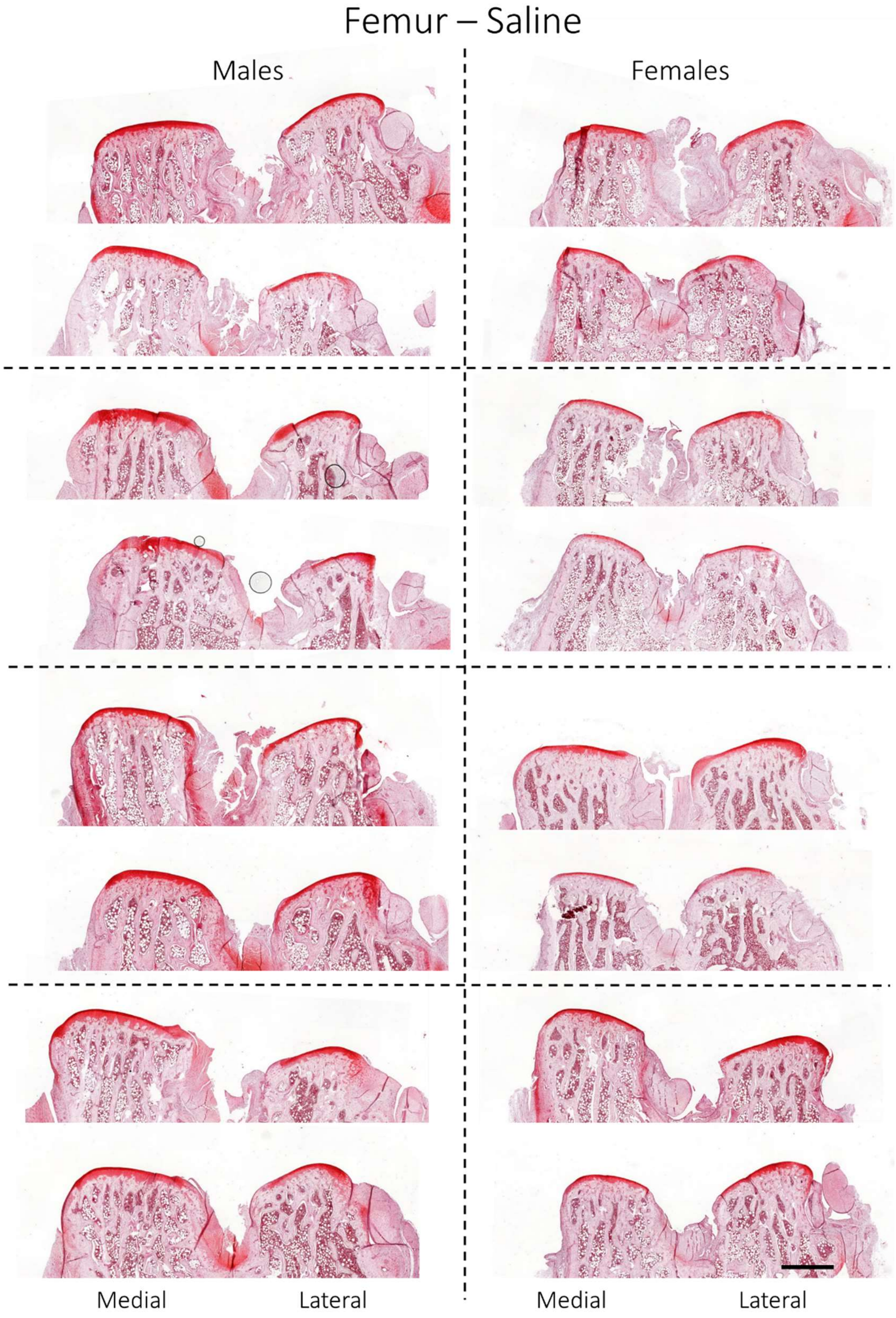

Figure S17: Complete safranin O histology of all the femurs in the saline group. Scale bar: 1 mm.

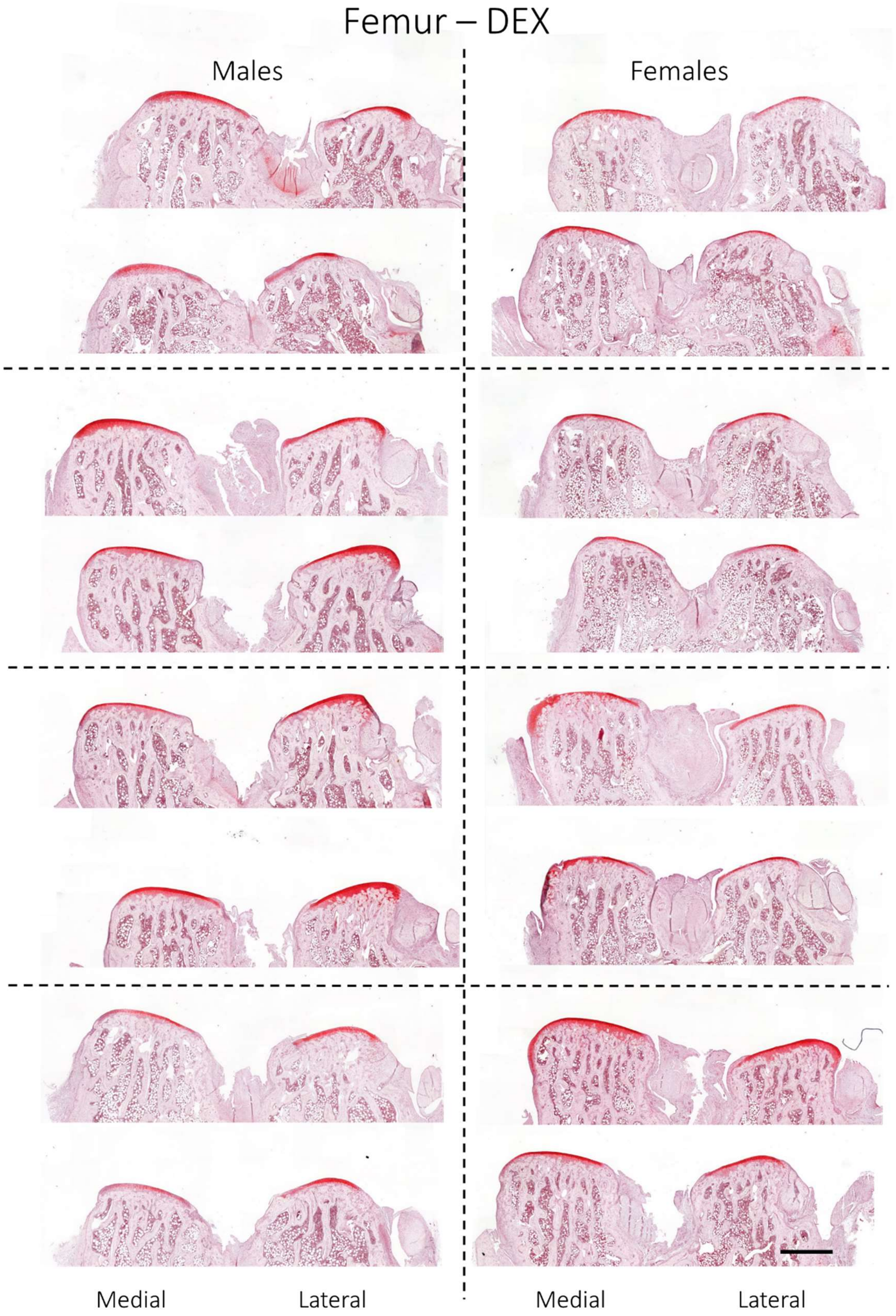

Figure S18: Complete safranin O histology of all the femurs in the DEX group. Scale bar: 1 mm.

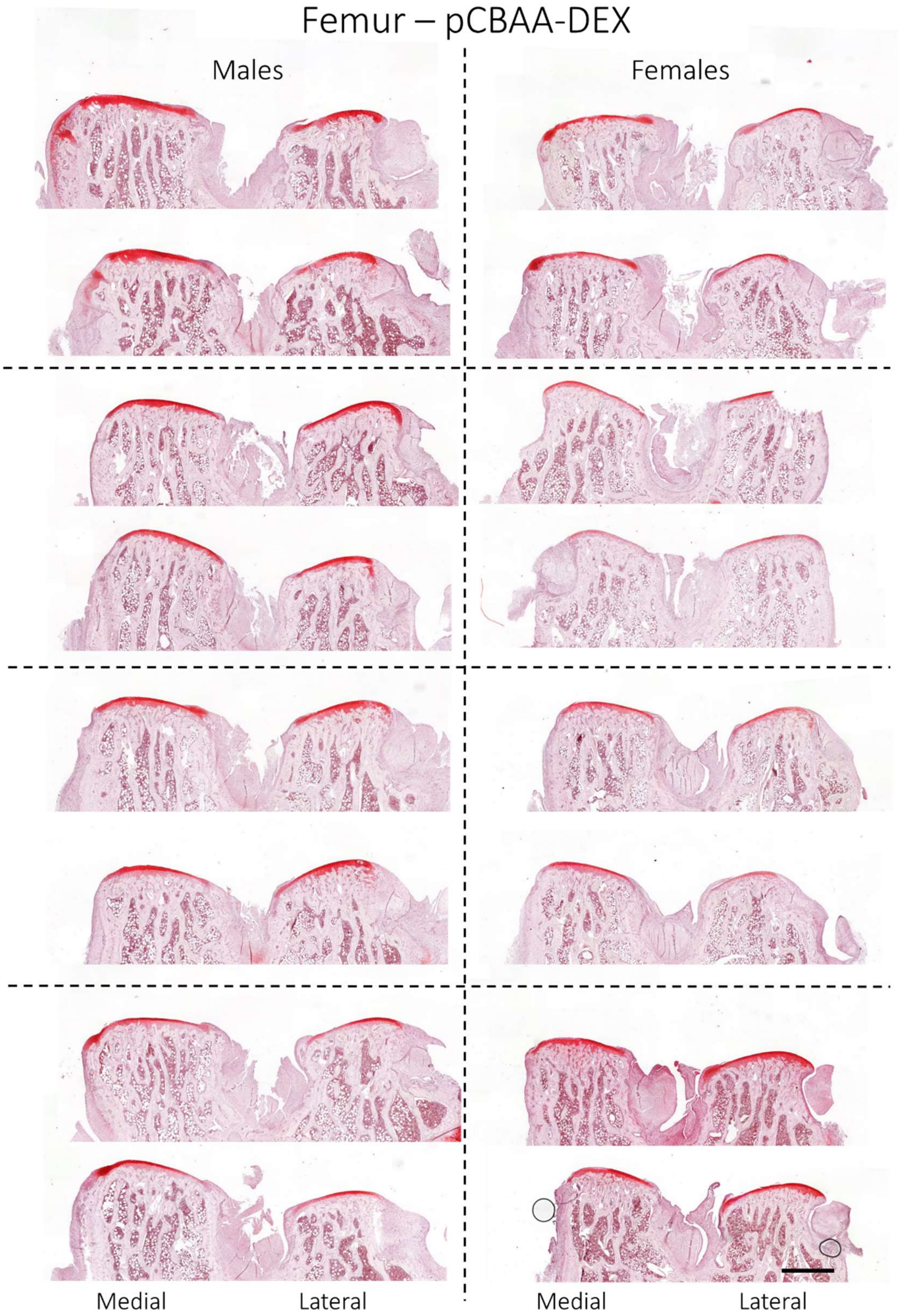

Figure S19: Complete safranin O histology of all the femurs in the pCBAA-DEX group. Scale bar: 1 mm.

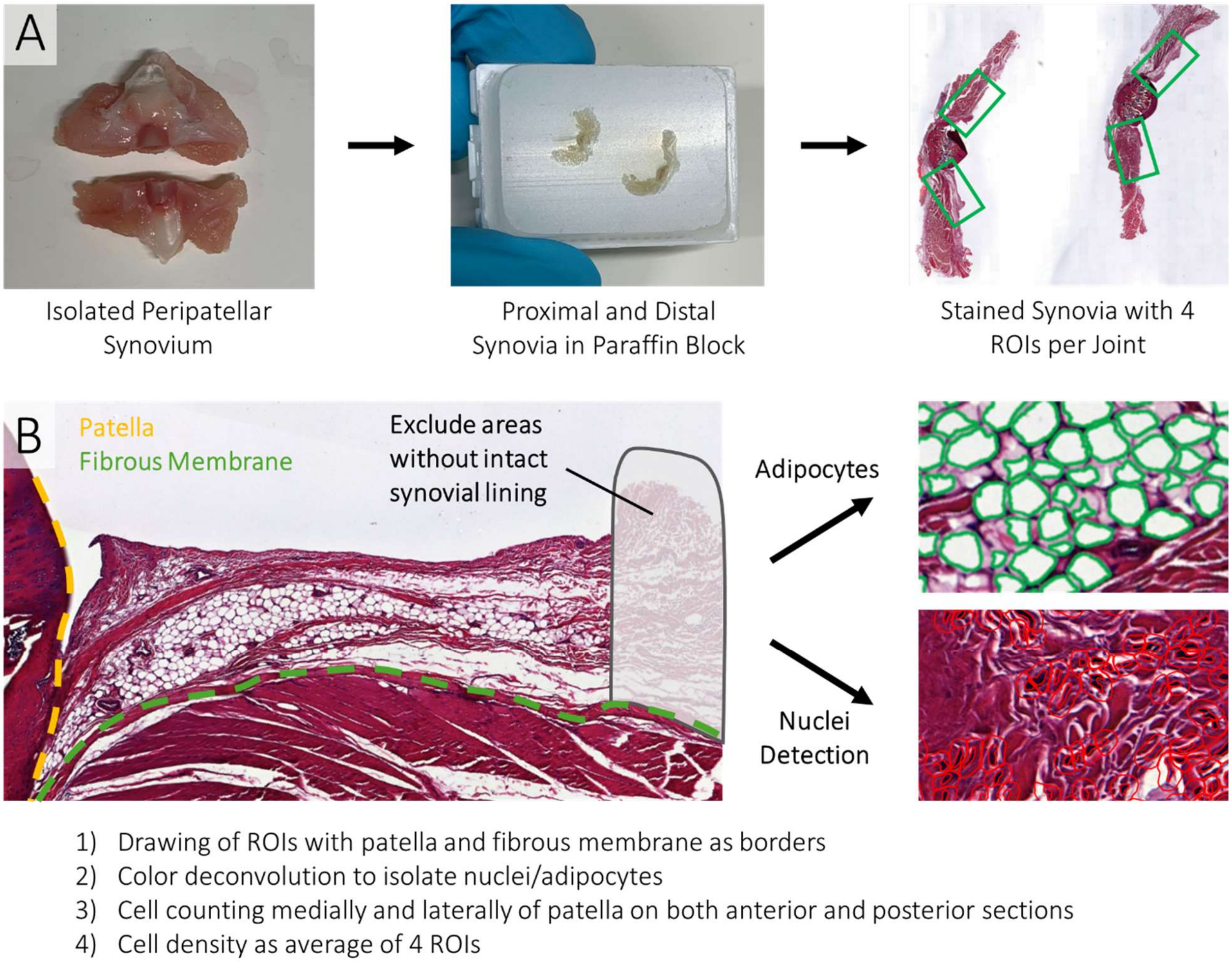

Figure S20: Synovium Cell Density Quantification: Schematic illustration of the methodology to process (A) and analyze (B) the peripatellar joint capsule to quantify the density of cells generally and adipocytes specifically. Figure was modified from Weber et al.(1)

A Cartilage Degeneration Score

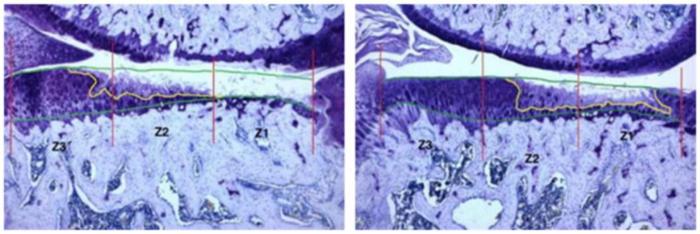

Table II. #2 Cartilage degeneration score

Parameter	Grade	Description
Cartilage degeneration	0	No degeneration
	1	Minimal degeneration; 5–10% of the total projected cartilage area affected by matrix or chondrocyte loss
	2	Mild degeneration; 11–25% affected
	3	Moderate degeneration; 26–50% affected
	4	Marked degeneration; 51–75% affected
	5	Severe degeneration; greater than 75% affected

B Total/Significant Cartilage Degeneration Width

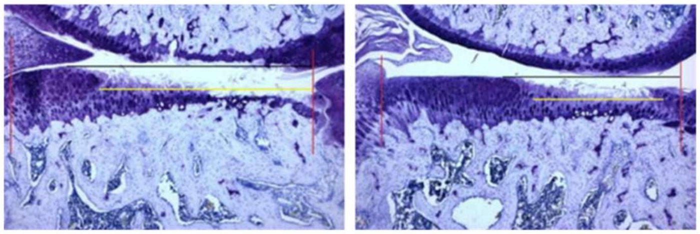

#3 and #4 Total and significant cartilage degeneration width. #3. The total cartilage degeneration width (black horizontal line) represents the total extent of the tibial plateau affected by any type of degeneration (matrix fibrillation/loss, PG loss with or without chondrocyte death). The measurement is taken at the projected cartilage surface from the outer edge of the tibial plateau, adjacent to the osteophyte (outer red line), to the point at which the cartilage is normal (inner red line). #4. The significant cartilage degeneration width (yellow horizontal line) represents the width of tibial cartilage in which 50% or more of the original cartilage thickness is seriously compromised by collagen matrix loss or loss of 50% of chondrocytes (and concurrent PG) loss. **A.** Example of tibial plateau with large total and significant tibial cartilage degeneration width and **B.** example with smaller cartilage degeneration width.

Figure S21: Cartilage histology grading: Illustrations reproduced from *Gerwin et al*. showing the methodology behind the CDS (A) and the TCDW/SCDW (B) gradings(2).

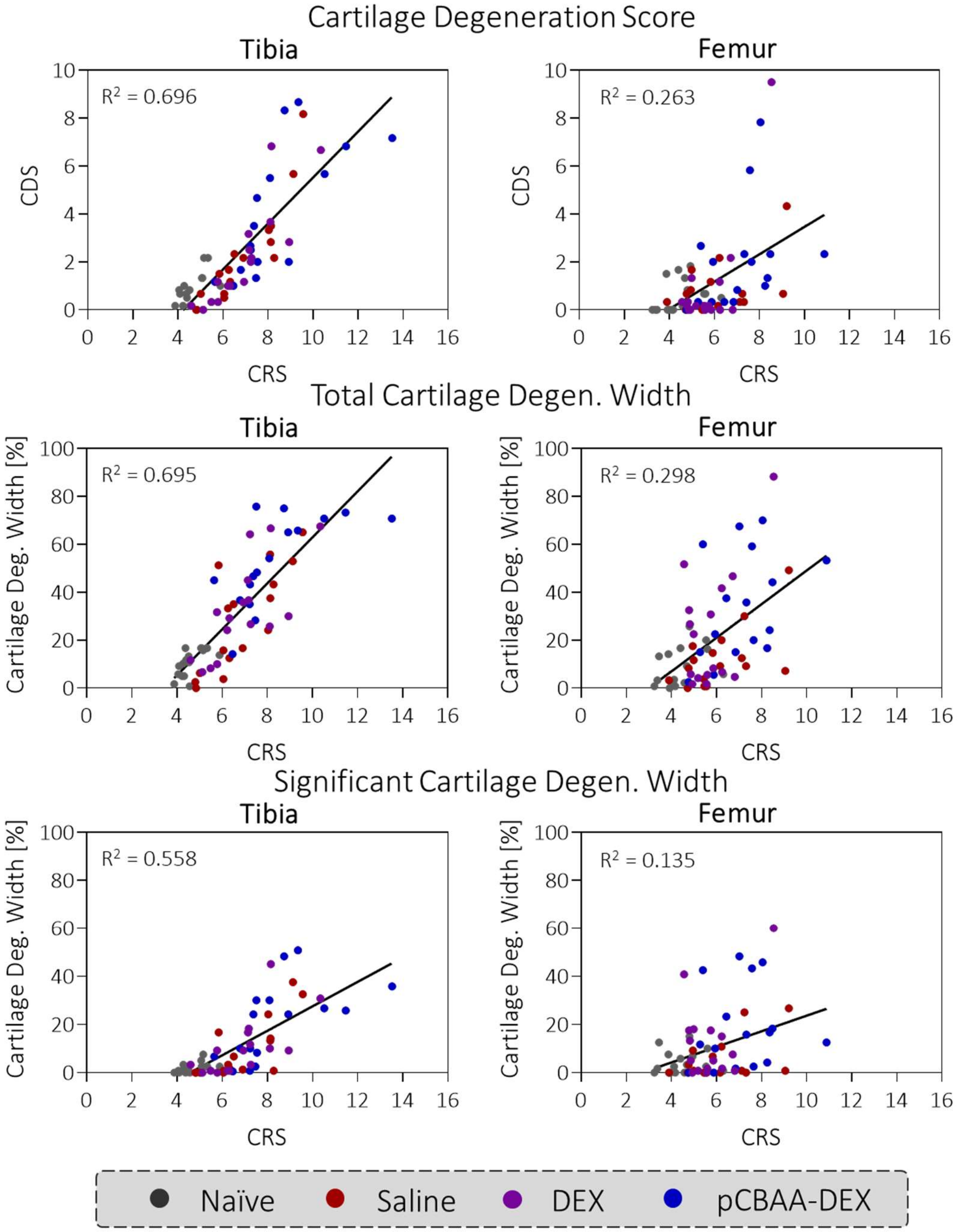

Figure S22: Correlation Plots of the CRS with Histology: Correlation analysis of the cartilage roughness score with the different metrics from the histology gradings yielded increased correlation coefficients for the tibia compared to the femur which is most probably due to the decreased magnitude of cartilage wear on the latter. Moreover, correlation with the surface sensitive cartilage degeneration score and total cartilage degeneration width were increased compared to the significant cartilage degeneration width that evaluates degeneration in the middle zone. Medial and lateral results are represented as individual datapoints.

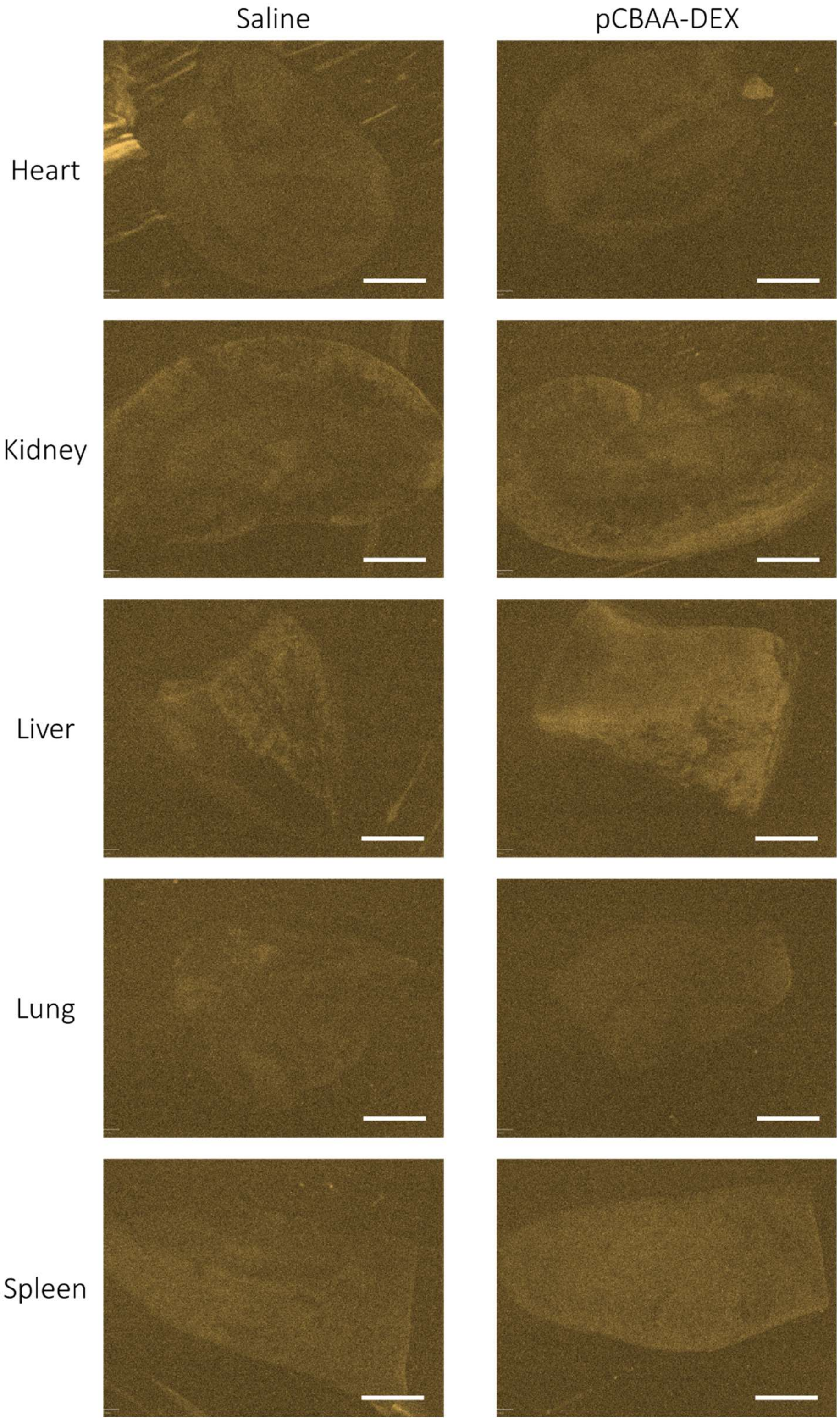

Figure S23: Fluorescence uptake in internal organs: Fluorescence stereomicroscopy images of internal organs showing no significant increase in fluorescence in any of the imaged organs in animals receiving intra-articular injections of fluorescent pCBAA-DEX. Scale bar: 5 mm.

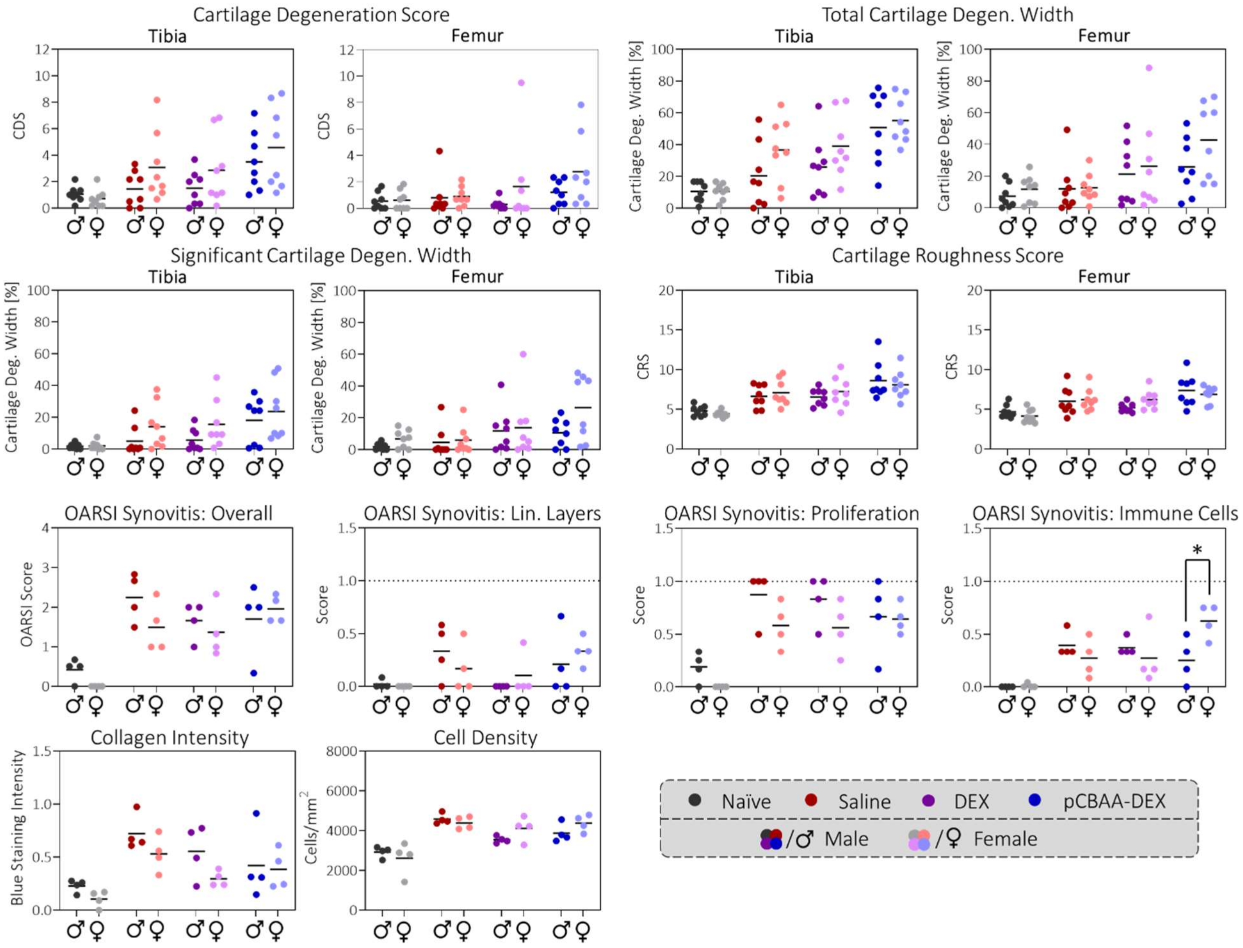

Figure S24: Sex-differences: Investigation of the sex differences in this study revealed a statistically non-significant trend towards increased levels of cartilage degeneration for the females compared to the males. Interestingly, this trend was however only visible for the histology gradings but not the CT-based cartilage roughness score. For the synovium, there is a trend towards increased severity in males vs. females for several investigated metrics. Note that for the cartilage metrics, the medial and lateral data is represented as separate datapoints.

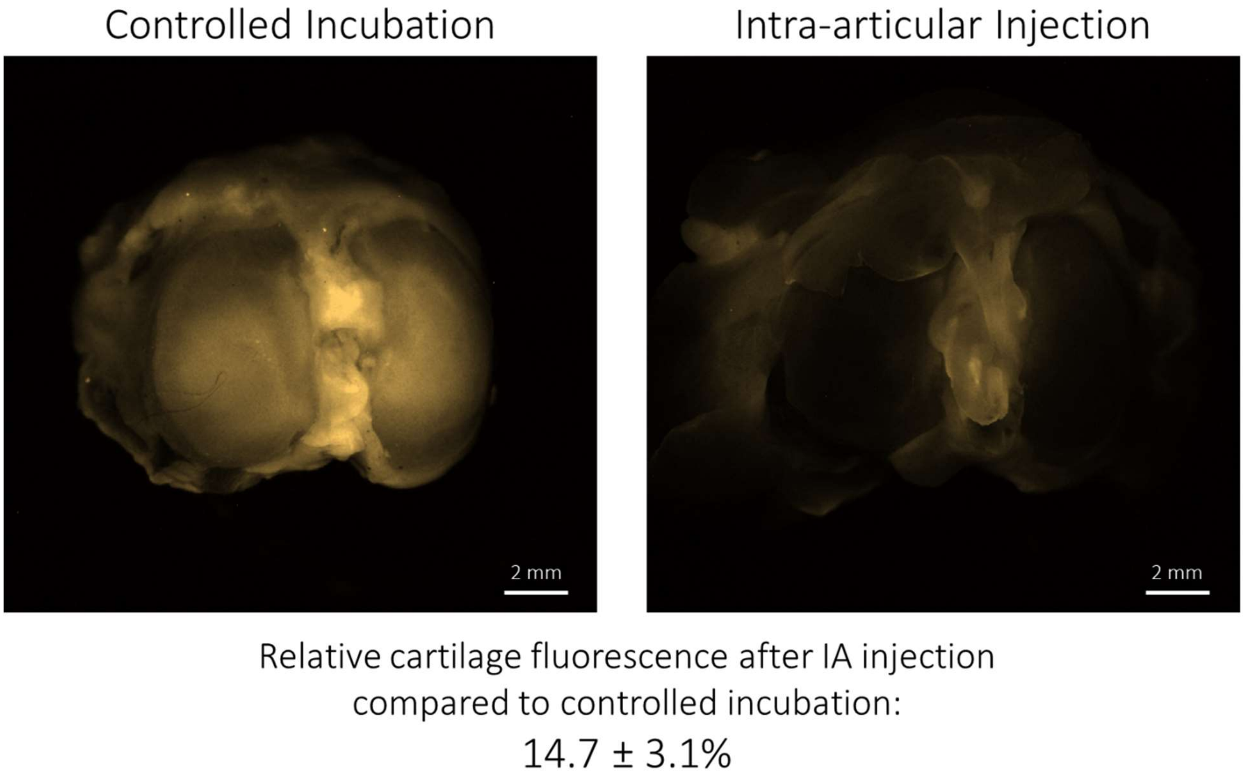

Figure S25: The cartilage fluorescence intensity is substantially decreased after intra-articular injection of pCBAA-DEX compared to the controlled incubation of cleaned-up tibias and femurs with the polymer solution.

References

1. Weber P, Bevc K, Fercher D, Kauppinen S, Zhang S, Asadikorayem M, et al. The collagenase-induced osteoarthritis (CIOA) model: Where mechanical damage meets inflammation. Osteoarthr Cartil Open [Internet]. 2024 [cited 2025 Jan 30];6(4):100539. Available from: https://www.sciencedirect.com/science/article/pii/S2665913124001067

2. Gerwin N, Bendele AM, Glasson S, Carlson CS. The OARSI histopathology initiative – recommendations for histological assessments of osteoarthritis in the rat. Osteoarthritis Cartilage [Internet]. 2010 Oct 1 [cited 2023 Mar 14];18:S24–34. Available from: https://www.sciencedirect.com/science/article/pii/S1063458410002475
